# Self-reported critical gaps in the essential knowledge and capacity of spatial epidemiology between the current university education and competency-oriented professional demands in preparing for a future pandemic among public health postgraduates in China: a nationwide cross-sectional survey

**DOI:** 10.1186/s12909-023-04578-6

**Published:** 2023-09-07

**Authors:** Tao Lan, Man Cheng, Yue-Dong Lin, Long-Yan Jiang, Ning Chen, Man-Tong Zhu, Qiao Li, Xian-Yan Tang

**Affiliations:** Department of Epidemiology and Biostatistics, School of Public Health, Guangxi Medical University, China. No. 22Nd, Shuangyong Road, Nanning, Guangxi Zhuang Autonomous Region 530021 People’s Republic of China

**Keywords:** Spatial epidemiology, Public health professionals, Public health postgraduates, University education, Competency-oriented professional demands

## Abstract

**Background:**

Spatial epidemiology plays an important role in public health. Yet, it is unclear whether the current university education in spatial epidemiology in China could meet the competency-oriented professional demands. This study aimed to understand the current situation of education and training, practical application, and potential demands in spatial epidemiology among public health postgraduates in China, and to assess the critical gaps in a future emerging infectious diseases (EID) pandemic preparedness and response.

**Methods:**

This study was divided into three parts. The first part was a comparative study on spatial epidemiology education in international public health postgraduate training. The second part was a cross-sectional survey conducted among public health professionals. The third part was a nationwide cross-sectional survey conducted among public health postgraduates at Chinese universities from October 2020 to February 2021. Data was collected by the WeChat-based questionnaire star survey system and analyzed using the SPSS software.

**Results:**

International education institutions had required public health postgraduates to master the essential knowledge and capacity of spatial epidemiology. A total of 198 public health professionals were surveyed, and they had a median of 4.00 (IQR 3.13–4.53) in demand degree of spatial epidemiology. A total of 1354 public health postgraduates were surveyed from 51 universities. Only 29.41% (15/51) of universities offered spatial epidemiology course. Around 8.05% (109/1354) of postgraduates had learned spatial epidemiology, and had a median of 1.05 (IQR 1.00–1.29) in learning degree and a median of 1.91 (IQR 1.05–2.78) in practical application degree of spatial epidemiology. To enhance professional capacity, 65.95% (893/1354) of postgraduates hoped that universities would deliver a credit-course of spatial epidemiology.

**Conclusions:**

A huge unmet education and training demand in spatial epidemiology existed in the current education system of public health postgraduates in China. To enhance the competency-oriented professional capacity in preparedness and response to a future pandemic, it is urgent to incorporate the teaching and training of spatial epidemiology into the compulsory curriculum system of public health postgraduates in China.

**Supplementary Information:**

The online version contains supplementary material available at 10.1186/s12909-023-04578-6.

## Background

Spatial epidemiology is an important disciplinary module for public health [1, 2]. With the rapid development of big data science and modern information technology, and the improvement of accessibility to health-related spatio-temporal big data, spatial epidemiology plays a more important role in public health [2–4].

Public health postgraduates will be the leading public health practitioners. In theory, university education and training of public health postgraduates should be oriented to the demands of public health fields, and be informed by international educational standards and educational practices of top-level universities worldwide [5, 6]. To enhance preparedness and response to a future pandemic of emerging infectious diseases (EID), public health postgraduates should manage the essential theory and knowledge of spatial epidemiology [7], and possess certain skills in identifying the spatio-temporal trend of diseases [8], detecting the spatio-temporal cluster of diseases [9], and conducting spatio-temporal risk assessment [10, 11]. Regarding international training programs for public health postgraduates, the education and cultivation of knowledge and capacity in spatial epidemiology are extremely highlighted. The Association of Schools of Public Health in the European Region (ASPHER) emphasized that public health postgraduates should predict the health status of populations using geographically relevant information [12]. The Association of School & Programs of Public Health (ASPPH) addressed the use of informatics methods and resources by public health postgraduates [13]. Furthermore, the curriculum systems of public health talent training in top-level universities worldwide also reflect the necessity and significance of public health postgraduates to master spatio-temporal skills [14].

Professional knowledge and capacity in spatial epidemiology are greatly demanded in public health fields [15]. Especially during the COVID-19 pandemic, these demands were even more significant and urgent. Integrating geographic information science and technology (GIS&T) into surveillance, modeling, and response to the COVID-19 pandemic has enhanced understanding and control of the disease compared to the SARS-COV outbreaks during 2002–2003 and MERS-CoV outbreaks during 2012–2014 [16]. Domains of integrating spatial analysis techniques into prevention and control against the COVID-19 pandemic included determining the spatio-temporal trajectory of patients [17], digitally tracing the personnel of close-contact exposure using geospatial technologies [18], and assessing the geographical-social susceptibilities of population and health disparities [19].Public health fields are increasingly turning toward using spatial analysis techniques [20]. Besides, WHO has put forward the transformation path of health data. Health-related data was visualized by spatial analysis techniques and transformed into knowledge to assist in making decisions for governments [21]. Successful applications of spatial epidemiology have been reported in public health fields [22–24]. These applications consistently highlighted the significance of spatial epidemiology in public health practice.

Under the international education trends and professional competencies requirements of public health fields for public health personnel, it is urgent to strengthen the training of public health postgraduates in spatial epidemiology during the university education period. University education, particularly in the regular and standardized curriculum education system, is the key approach to cultivating the professional thinking and capacity of spatial epidemiology among public health postgraduates [7]. However, it is unclear whether the current situation of university education and training in spatial epidemiology among public health postgraduates in China could meet the capacity reserve and talent preparation needed to respond to a future pandemic of EID, and whether a huge gap existed between the current situation of university education and the demands of public health fields. Therefore, we conducted this study to compare spatial epidemiology education in international public health postgraduate training, to assess the professional demands for spatial epidemiology among public health fields, to identify the current situation of university education and training in spatial epidemiology and the learning, application, and potential demand of public health postgraduates for spatial epidemiology in China, and then to assess the critical gaps in a future EID pandemic preparedness and response. This study would provide evidence for refining the training mode of public health talents in Chinese universities, particularly in enhancing the professional capacity of spatial epidemiology and spatio-temporal big data science to cope with a future pandemic of EID.

## Methods

### Study setting

The development situation of public health postgraduate education system in China is shown in Fig. S1. At present, 155 universities in China have set up public health education, 96 universities offer master-degree education in public health, and 36 universities offer PhD-degree education in public health. Specifically, 29 national-level universities and 67 non-national-level universities offer master-degree education in public health [25]. The curriculum system of public health postgraduates in Chinese universities consists of four parts, i.e., public compulsory courses, specialized core courses, specialized compulsory courses, and elective courses. Furthermore, specialized core courses include biostatistics, epidemiology, public health pedagogy, etc. [26].

### Study design and study population

This study was divided into three parts. The first part was a comparative study on spatial epidemiology education in international public health postgraduate training. The second part was a cross-sectional survey conducted among public health professionals at 15 municipal and provincial CDCs in Guangxi, China from March 2023 to April 2023. The third part was a national cross-sectional survey conducted among public health postgraduates in Chinese universities from October 2020 to February 2021.

International education institutions include the Association of Schools of Public Health in the European Region (ASPHER) and the Association of School & Programs of Public Health (ASPPH). The top-level universities worldwide include Johns Hopkins University, London School of Hygiene and Tropical Medicine, Yale University, Harvard University, University of Michigan, and Boston University.

The public health professionals included in this study should be (1) from 15 municipal and provincial CDCs in Guangxi, China, (2) from key sections of public health practice, and (3) the chief or deputy chief of these sections.

The public health postgraduates included in this study should be (1) registered as master-degree postgraduates and PhD-degree postgraduates, and (2) enrolled in universities recognized by the Chinese Ministry of Education.

### Sampling technique

Public health professionals were recruited based on purpose sampling, and public health postgraduates were recruited based on convenience sampling.

### Questionnaire development

Two self-designed online questionnaires were developed to measure the demands for spatial epidemiology among public health professionals and the professional knowledge and capacity of spatial epidemiology among public health postgraduates in China. We completed the draft of questionnaires after reviewing the literature and consulting with relevant experts. Based on the pre-survey, the drafts of the questionnaires were modified and improved, The contents of the final questionnaires are presented in Supplementary Material 1 and Supplementary Material 2.

### Measurement

The learning degree of spatial epidemiology of public health postgraduates was scored on a 3-point Likert scale. The practical application degree, efficacy perception degree, and demands degree of spatial epidemiology of public health postgraduates were scored on a 5-point Likert scale. The calculations of the learning degree, practical application degree, efficacy perception degree, and demands degree of public health professionals and public health postgraduates are presented in Supplementary Material 3.

### Data collection and data management

The educational materials on spatial epidemiology in international public health postgraduate training were searched from the official websites of education international institutions and top-level universities worldwide. The questionnaire data was collected via the WeChat online questionnaire star survey system (Ranxing Information Technology Company, Limited). The key informants sent the filling link of online questionnaire to the study populations via WeChat (Tencent Holdings Limited). Eligible study populations were invited to fill out the questionnaire. Finally, we exported the data from the questionnaire star system and developed an Excel database to manage the data.

### Quality control

Prior to survey, expert consultation was implemented during questionnaire development and pilot study. During the survey, we controlled the quality of questionnaires completion by (1) contacting the director or deputy director of the CDC to be the unit's key informant, and contacting the dean or deputy dean of the university's school of public health to be the university's key informant, (2) clarifying the scope and the object of survey, (3) sending the filling links of online questionnaires to eligible study population by the key informants, and (4) tracking the questionnaire. We set up the following quality controls during the data collection process: (1) verification code to prevent repeated submissions, (2) integrity restrictions to avoid incomplete questionnaires, and (3) answer time of at least 120 s to avoid invalid questionnaires. Post the survey, the questionnaire data was cleaned and the unqualified questionnaires were eliminated.

### Data analysis

Statistical analysis was conducted using SPSS (version 22). Frequency and percentage were used to statistically describe categorical data. The normality of continuous data was assessed using the Shapiro–Wilk test. As data was not distributed normally, the median and interquartile range (IQR) were used for statistical description. Comparisons of the learning degree, practical application degree, efficacy perception degree, and potential demands degree in spatial epidemiology were performed using the Mann–Whitney U test between postgraduates at national-level universities and non-national-level universities, between master-degree and PhD-degree postgraduates, between science-degree and professional-degree postgraduates, respectively. Comparisons of the learning degree, practical application degree, efficacy perception degree, and potential demands degree in spatial epidemiology were performed using the Kruskal–Wallis test among postgraduates majored in different second-level disciplines of public health, and among postgraduates graded in different academic years. Comparison of categorical data between groups was conducted using the chi-square test. P-value less than 0.05 was considered as significant. The thematic map was produced using ESRI ArcGIS software (version 10.7).

## Results

### The situation of sptial epidemiology education in international public health postgraduate training

The ASPHER and ASPPH had developed training standards for the essential knowledge and capacity in spatial epidemiology required for public health postgraduates (Table S1). Top-level universities worldwide such as the London School of Hygiene and Tropical Sciences had offered spatial epidemiology relevant courses for public health postgraduates (Table S2).

### The situation of learning, practical application, and professional demands of spatial epidemiology among public health professionals

A total of 229 public health professionals were invited to participate, and 198 public health professionals effectively completed the survey (participation rate: 86.46%). Only 38.38% (76/198) of public health professionals had learned spatial epidemiology, and 19.70% (39/198) had applied spatial epidemiology. However, public health professionals had a high median of 4.00 (IQR 3.83-4.67) (total score of 5) on the efficacy perception of spatial epidemiology, and had a high demands degree in spatial epidemiology, with a median of 4.00 (IQR 3.13–4.53) (total score of 5) (Table 1).

**Table 1:** The average score of public health professionals on the degrees of theoretical learning, practical application, efficacy perception, and demand for spatial epidemiology (median, IQR)

Items	Median	IQR	Full mark
Degree of learning in different subsections	1.00	1.00–1.38	3.00
Subsection of study design theories	1.00	1.00–1.50	3.00
Subsection of spatio-temporal data analysis methods	1.00	1.00–1.22	3.00
Degree of applying in different subsections	1.71	1.43–2.27	5.00
Subsection of study design theories	2.00	1.50–2.50	5.00
Subsection of spatio-temporal data analysis methods	2.00	1.38–2.38	5.00
Subsection of spatio-temporal statistical analysis software	1.31	1.15–2.00	5.00
Degree of efficacy perception	4.00	3.83–4.67	5.00
Degree of professional demands	4.00	3.13–4.53	5.00

### The perceptions of public health professionals on public health postgraduate study spatial epidemiology

Around 90.91% of public health professionals believed that public health postgraduates should learn spatial epidemiology. The degree of necessity that public health professionals perceived that public health postgraduates should learn spatial epidemiology was high, with a median of 4.00 (IQR 3.86–4.00) (total score of 5) (Table S3).

### The situation of education and training in spatial epidemiology at universities

**Fig. 1:**
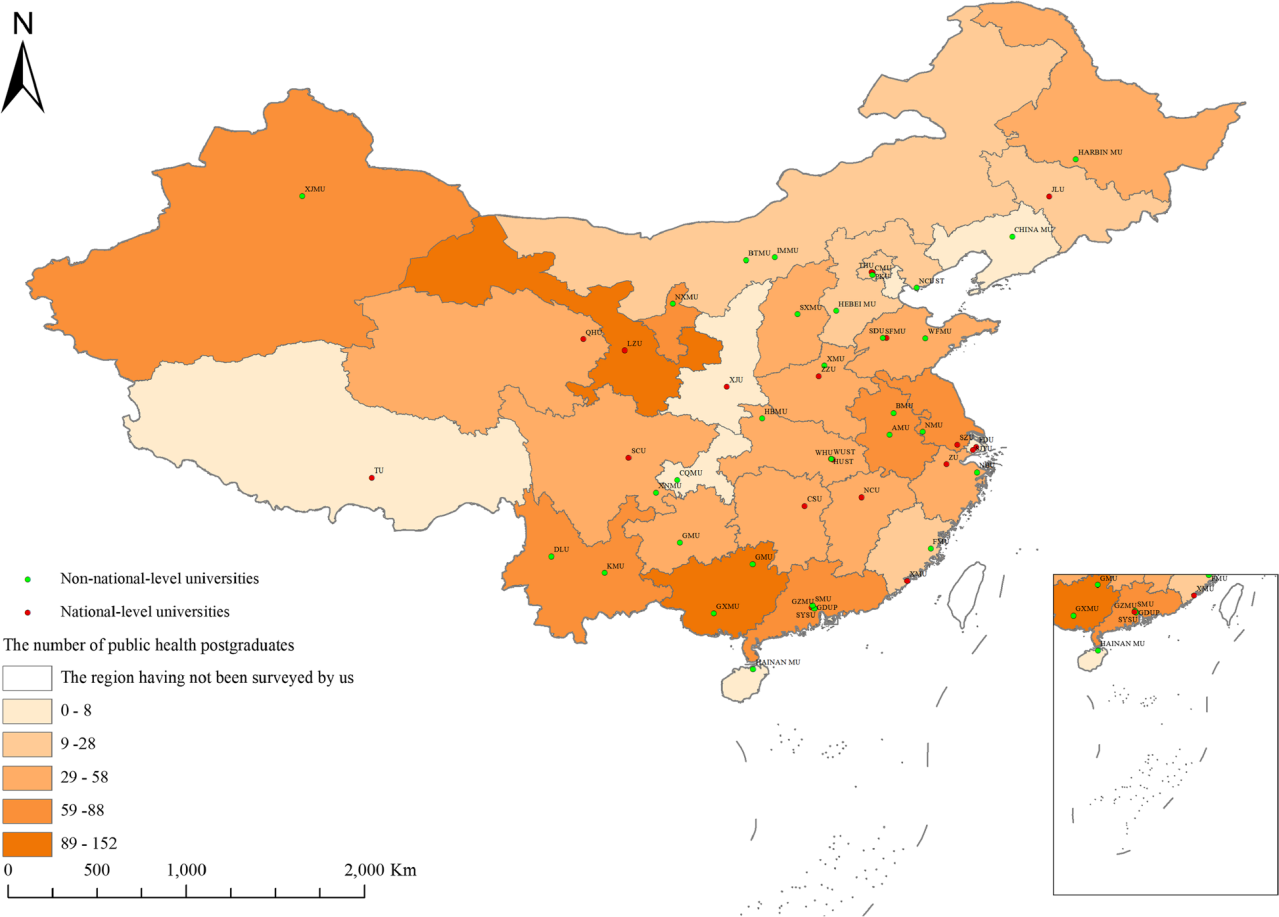
The universities and public health postgraduates surveyed in China

A total of 51 universities were surveyed, including 20 national-level universities and 31 non-national-level universities. The geographical distribution of surveyed universities and public health postgraduates is shown in Fig. 1. Only 29.41% (15/51) of universities offered education and training in spatial epidemiology. The proportion of offering spatial epidemiology course was 20% (4/20) at national-level universities and 35.48% (11/31) at non-national-level universities. There was no statistical significance in offering the course between national-level universities and non-national-level universities (Table S4). Universities offering the course were mainly located in eastern, southwestern, and northwestern China (Fig. S2).

### The situation of learning of spatial epidemiology among public health postgraduates

A total of 1501 public health postgraduates in China were invited to participate, and 1354 postgraduates effectively completed the survey (participation rate: 90.21%). Only 8.05% (109/1354) of postgraduates had been educated and trained in spatial epidemiology (Table 2). And 31.19% (34/109) learned spatial epidemiology through university-offering courses (Table S5). The proportion of having learned spatial epidemiology was 10.28% (47/457) for postgraduates at national-level universities, significantly higher than that 6.91% (62/897) for postgraduates at non-national-level universities (*P* < 0.05, Table 2). Between the second-level disciplines of public health, 20.83% (5/24) postgraduates from the discipline of maternal-child-and-adolescent hygiene had learned spatial epidemiology, higher than that from other second-level disciplines (*P* < 0.05, Table 2). Around 13.33% (42/315) of postgraduates in the second academic year had learned spatial epidemiology, higher than those in other academic years (*P* < 0.05, Table 2).

**Table 2:** The learning situation of spatial epidemiology among public health postgraduates (n, %)

Variable	Total	Status of having learned the course of spatial epidemiology		*P-*value^#^
		**Yes**	**No**	
**Public health postgraduate students**	1354 (100.00)	109 (8.05)	1245 (91.95)	
**Levels of universities**				
National-level universities	457 (34.00)	47 (10.28)	410 (89.72)	**0.031**
Non-national-level universities	897 (66.00)	62 (6.91)	835 (93.09)	
**Candidates' degrees**				
Master-degree public health postgraduates	1250 (92.32)	101 (8.08)	1149 (91.92)	0.889
PhD-degree public health postgraduates	104 (8.68)	8 (7.69)	96 (92.31)	
**Types of degrees**				
Science-degree public health postgraduates	694 (51.26)	56 (8.07)	638 (91.93)	0.979
Professional-degree public health postgraduates	660 (48.74)	53 (8.03)	607 (91.97)	
**Second-level disciplines of public health**				
Public health (i.e., MPH and DrPH)	660 (48.74)	53 (8.03)	607 (91.97)	** < 0.001**
Epidemiology and health statistics	348 (25.70)	41(11.78)	307 (88.22)	
Health toxicology	44 (3.25)	2 (4.55)	42 (95.45)	
Occupational and environmental hygiene	90 (6.65)	5 (5.56)	85 (94.44)	
Nutrition and food hygiene	59 (4.36)	2 (3.39)	57 (96.61)	
Maternal-child-and-adolescent hygiene	24 (1.77)	5 (20.83)	19 (79.17)	
Social medicine and health management	101 (7.46)	1 (0.99)	100 (99.01)	
Other second-level disciplines	28 (2.07)	0 (0.00)	28 (100.00)	
**Academic years**				
Postgraduates in the first academic year	761 (56.20)	35 (4.60)	726 (95.40)	** < 0.001**
Postgraduates in the second academic year	315 (23.26)	42 (13.33)	273 (86.67)	
Postgraduates in the third academic year	230 (16.99)	27 (11.74)	203 (88.26)	
Postgraduates in the fourth or above academic year	48 (3.55)	5 (10.42)	43 (89.58)	

^#^Frequency and percentage were used for statistical description and the comparison was conducted by Chi-square test

Public health postgraduates had limited knowledge of spatial epidemiology, and the median was 1.05 (IQR 1.00–1.29) (total score of 3). The learning degree was non-significant between postgraduates at national-level universities and non-national-level universities, between master-degree and PhD-degree postgraduates, between science-degree and professional-degree postgraduates, among postgraduates majored in different second-level disciplines of public health, and among postgraduates graded in different academic years (*P* > 0.05, Tables S6, S7, S8, S9, S10).

### The situation of practical application of spatial epidemiology among public health postgraduates

Public health postgraduates had limited practical application of spatial epidemiology, and the median was 1.91 (IQR 1.05–2.78) (total score of 5). The practical application degree was non-significant between postgraduates at national-level universities and at non-national-level universities, between master-degree and PhD-degree postgraduates, between science-degree and professional-degree postgraduates, among postgraduates majored in different second-level disciplines of public health, and among postgraduates graded in different academic years (*P* > 0.05, Tables S6, S7, S8, S9, S10).

### Efficacy perception of application of spatial epidemiology to solve public health issues

Overall, public health postgraduates having learned spatial epidemiology had a high median of 4.00 (IQR 3.67–4.33) (total score of 5) on the efficacy perception of the application of spatial epidemiology to solve public health issues (Table 3). Notably, postgraduates at national-level universities perceived more importance of spatial epidemiology to solve public health issues (median 4.00, IQR 3.83–4.67) than those at non-national-level universities (median 4.00, IQR 3.63–4.04) (*P* < 0.05, Table 3). PhD-degree postgraduates perceived more importance of spatial epidemiology to solve public health issues (median 4.17, IQR 4.00–5.00) than master-degree postgraduates (median 4.00, IQR 3.67–4.33) (*P* < 0.05, Table 3).

**Table 3:** The median on the degree in efficacy perception of spatial epidemiology among public health postgraduates

Variable	n (%)	Median (IQR)	*P*-value
**Public health postgraduates having learned spatial epidemiology**	109 (100.00)	4.00 (3.67–4.33)	
**Levels of universities**			
National-level universities	47 (43.12)	4.00 (3.83–4.67)	**0.033**^**#**^
Non-national-level universities	62 (58.88)	4.00 (3.63–4.04)	
**Candidates' degrees**			
Master-degree public health postgraduates	101 (92.66)	4.00 (3.67–4.33)	**0.020**^**#**^
PhD-degree public health postgraduates	8 (7.34)	4.17 (4.00–5.00)	
**Types of degrees**			
Science-degree public health postgraduates	56 (51.38)	4.00 (3.67–4.29)	0.863^#^
Professional-degree public health postgraduates	53 (48.62)	4.00 (3.67–4.50)	
**Second-level disciplines of public health**			
Public health (i.e., MPH and DrPH)	53 (48.62)	4.00 (3.67–4.50)	0.853^†^
Epidemiology and health statistics	41 (37.61)	4.00 (3.58–4.42)	
Other second-level disciplines	15 (13.76)	4.00 (3.83–4.17)	
**Academic years**			
Postgraduates in the first academic year	35 (32.11)	4.00 (3.67–4.17)	0.005^†^
Postgraduates in the second academic year	42 (38.53)	4.00 (3.42–4.00)	
Postgraduates in the third or above academic year	32 (29.56)	4.25 (3.83–4.96)	

^†^Median and interquartile range (IQR) were used for statistical description and the comparison was conducted by Kruskal–Wallis test

^#^Median and interquartile range (IQR) were used for statistical description and the comparison was conducted by Mann–Whitney U test

### Potential demands in spatial epidemiology among public health postgraduates

Overall, public health postgraduates had a high degree of potential demands in spatial epidemiology (Table 4), with a median of 3.33 (IQR 2.67–4.00) (total score of 5). The degree of potential demands in spatial epidemiology was high among public health postgraduates in most provinces of China (Fig. S3). Notably, PhD-degree postgraduates had stronger demands in spatial epidemiology (median 3.83, IQR 2.54–4.17) than master-degree postgraduates (median 3.33, IQR 2.67–4.00) (*P* < 0.05, Table 4). Postgraduates who majored in epidemiology and health statistics had stronger demands in spatial epidemiology (median 3.67, IQR 2.83–4.00) than those who majored in public health (median 3.33, IQR 2.83–4.00) and other second-level disciplines (median 3.00, IQR 2.17–4.00) (*P* < 0.05, Table 4).

**Table 4:** The median on the degree of demands in spatial epidemiology among public health postgraduates

Variable	n (%)	Median (IQR)	*P-*value
**Public health postgraduates**	1354 (100.00)	3.33 (2.67–4.00)	
**Levels of universities**			
National-level universities	457 (33.75)	3.33 (2.67–4.00)	0.765^#^
Non-national-level universities	897 (66.25)	3.33 (2.67–4.00)	
**Candidates' degrees**			
Master-degree public health postgraduates	1250 (92.32)	3.33 (2.67–4.00)	**0.031**^**#**^
PhD-degree public health postgraduates	104 (7.68)	3.83 (2.54–4.17)	
**Types of degrees**			
Science-degree public health postgraduates	694 (51.26)	3.33 (2.50–4.00)	0.264^#^
Professional-degree public health postgraduates	660 (48.74)	3.33 (2.83–4.00)	
**Second-level disciplines of public health**			
Public health (i.e., MPH and DrPH)	660 (48.74)	3.33 (2.83–4.00)	** < 0.001**^**†**^
Epidemiology and health statistics	348 (25.70)	3.67 (2.83–4.00)	
Other second-level disciplines	246 (18.16)	3.00 (2.17–4.00)	
**Academic years**			
Postgraduates in the first academic year	761 (56.20)	3.33 (2.75–4.00)	0.222^†^
Postgraduates in the second academic year	315 (23.26)	3.17 (2.33–4.00)	
Postgraduates in the third or above academic year	278 (20.53)	3.50 (2.83–4.00)	

^†^Median and interquartile range (IQR) were used for statistical description and the comparison was conducted by Kruskal–Wallis test

^#^Median and interquartile range (IQR) were used for statistical description and the comparison was conducted by Mann–Whitney U test

Around 65.95% (893/1354) public health postgraduates perceived that universities should offer relevant courses in spatial epidemiology (Table 5). Specifically, postgraduates at national-level universities had stronger demands in spatial epidemiology course than those at non-national-level universities (69.15% vs. 64.33%, *P* < 0.05, Table 5). Compared with postgraduates in other academic years, postgraduates in the third academic year (76.96%, 177/230) were more eager to hope universities offer spatial epidemiology course (*P* < 0.05, Table 5).

**Table 5:** The demands in offering the course of spatial epidemiology among public health postgraduates (n, %)

Variable	Overall	The necessity of offering the course of spatial epidemiology at universities			*P-* value^#^
		**Necessary**	**Not sure**	**Not necessary**	
**Public health postgraduates**	1354 (100.00)	893 (65.95)	443 (32.72)	18 (1.33)	
**Levels of universities**					
National-level universities	457 (33.75)	316 (69.15)	128 (28.01)	13 (2.84)	** < 0.001**
Non-national-level universities	897 (66.25)	577 (64.33)	315 (35.12)	5 (0.56)	
**Candidates' degrees**					
Master-degree public health postgraduates	1250 (92.32)	818 (65.44)	416 (33.28)	16 (1.28)	0.284
PhD-degree public health postgraduates	104 (7.68)	75 (72.12)	27 (25.96)	2 (1.92)	
**Types of degrees**					
Science-degree public health postgraduates	694 (51.26)	466 (67.15)	219 (31.56)	9 (1.30)	0.636
Professional-degree public health postgraduates	660 (48.74)	427 (64.70)	224 (33.94)	9 (1.36)	
**Second-level disciplines of public health**					
Public health (i.e., MPH and DrPH)	660 (48.74)	427 (64.70)	224 (33.94)	9 (1.36)	0.061
Epidemiology and health statistics	348 (25.70)	253 (72.70)	92 (26.44)	3 (0.86)	
Health toxicology	44 (3.25)	24 (54.55)	19 (43.18)	1 (2.27)	
Occupational and environmental hygiene	90 (6.65)	63 (70.00)	25 (27.78)	2 (2.22)	
Nutrition and food hygiene	59 (4.36)	33 (55.93)	25 (42.37)	1 (1.69)	
Maternal-child-and-adolescent hygiene	24 (1.77)	15 (62.50)	9 (37.50)	0 (0.00)	
Social medicine and health management	101 (7.46)	66 (65.35)	33 (32.67)	2 (1.98)	
Other second-level disciplines	28 (2.07)	12 (42.86)	16 (57.14)	0 (0.00)	
**Academic years**					
Postgraduates in the first academic year	761 (56.20)	455 (59.79)	296 (38.90)	10 (1.31)	** < 0.001**
Postgraduates in the second academic year	315 (23.26)	227 (72.06)	83 (26.35)	5 (1.59)	
Postgraduates in the third academic year	230(16.99)	177(76.96)	51(22.17)	2(0.87)	
Postgraduates in the fourth or above academic year	48(3.55)	34(70.83)	13(27.08)	1(2.08)	

^#^Frequency and percentage were used for statistical description and the comparison was conducted by Chi-square test

## Discussion

Our study found that international education institutions had developed training standards for essential knowledge and capacity of spatial epidemiology required for public health postgraduates, and top-level universities worldwide had already offered corresponding courses to cultivate these essential knowledge and capacity of spatial epidemiology. There was a great professional demand in the public health fields for spatial epidemiology. The proportion of offering spatial epidemiology course was limited at Chinese universities. Public health postgraduates had limited knowledge of spatial epidemiology and limited practical application of spatial epidemiology. However, public health postgraduates had high potential demands in spatial epidemiology. Furthermore, we identified critical gaps in the essential knowledge and capacity of spatial epidemiology between the current university education and competency-oriented professional demands in preparing for a future pandemic among public health postgraduates in China.

The limited learning-and-practical application degrees of spatial epidemiology among public health postgraduates might be related to the limited proportion of offering spatial epidemiology course at universities. This study showed that public health postgraduates rarely learned the essential theory, knowledge, and capacity of spatial epidemiology through systematic courses provided by universities. A previous study found that public health postgraduates lacked of spatial epidemiology thinking during the COVID-19 outbreak in China, mainly due to the absence of university education in spatial epidemiology [27]. In theory, national-level universities are the key universities for training talents [28]. There was no statistical significance in offering spatial epidemiology course between national-level universities and non-national-level universities. This fact might suggest that education and training in spatial epidemiology were not greatly highlighted in China. Moreover, the learning-and-application degrees of spatial epidemiology were non-significant between master-degree and PhD-degree postgraduates, between science-degree and professional-degree postgraduates, among postgraduates majored in different second-level disciplines of public health, and among postgraduates graded in different academic years. This situation implied that the distinction degree of the curriculum system had not been considerably achieved in China [26].

The learning-and-application degrees of spatial epidemiology among public health postgraduates did not reach the requirements of public health talent storage. In 2013, the Chinese Ministry of Education proposed that public health postgraduates should systematically master the relevant interdisciplinary knowledge [29]. The related interdisciplinary knowledge included big data science (e.g., spatio-temporal big data), statistical method application, and biological information. In addition to the competencies required for the master-degree public health postgraduates, the PhD-degree public health postgraduates were required to have in-depth learning of frontier theories and methods of the major. As a new frontier inter-discipline, spatial epidemiology plays a major role in public health practices [30]. However, Combining the international educational trends and professional demands in public health fields, we found the deficiencies and inadequacies in university education in spatial epidemiology among public health postgraduates in China, and the situation of learning and application in spatial epidemiology among public health postgraduates were severe.

After the COVID-19 outbreak, the concept of returning to social model ‘Public Health 3.0’ was put forward internationally. It emphasizes that public health sectors should collaborate with agencies outside of the healthcare organization, such as bureau of health-related big data [31]. The Ministry of Education carried out the training mode reform of inter-disciplinary public health talents, strengthening the training in spatio-temporal big data and data science for high-level applied public health talents [25]. Specifically, there are huge gaps between the current university education in spatial epidemiology among public health postgraduates in China and capacity reserve and talent preparation needed to respond to a future pandemic of EID.

Causes underlying the gaps were multifactor. First, the curriculum system of education and training programs at Chinese universities is not completely consistent with international standards. Spatial epidemiology course is commonly taught in the education program for public health postgraduates in western countries [32, 33]. Similar findings were revealed in Spain [34], for example, causal inference was added into the curriculum system of public health postgraduate education. Second, training and education in spatial epidemiology were launched relatively late in China, and there may be a shortage of specialized teachers well-trained in spatial epidemiology at Chinese universities [35]. Third, it is not deeply interdisciplinary between traditional epidemiology and geographic information science, as well as spatio-temporal big data science [27]. Thus there is still a paucity of epidemiologic research integrating of geographic perspectives [36].

It is encouraged that public health postgraduates have perceived the importance of spatial epidemiology. Specifically, postgraduates who majored in epidemiology and health statistics had stronger demands in spatial epidemiology, as epidemiology and health statistics was a second-level discipline focusing on the cultivation of theoretical methods [37]. Consistent with findings in other studies [7, 38], public health talents had realized the importance of learning spatial epidemiology, and perceived the necessity of university education and training in spatial epidemiology in China.

### Strengths and limitations

This study had several strengths. First, public health postgraduates were from various universities across China, and the findings were well-generalized. Second, this study systematically examined whether there were critical gaps in a future EID pandemic preparedness and response by exploring the international education trends, the demands of public health fields, and the current situation of spatial epidemiology education in China. Despite the above strengths, limitations should be noted. As the questionnaires were distributed online, bias might exist.

### Policy implications

To enhance the competency-oriented professional capacity in preparedness and response to a future pandemic, this study has important implications for university education in spatial epidemiology among public health postgraduates. First, the demands in spatial epidemiology among public health professionals and public health postgraduates were large. Chinese universities should incorporate education and training in spatial epidemiology into the education system for public health postgraduates, particularly into the compulsory curriculum system, and enrich the teaching contents of spatial epidemiology course. Second, spatial epidemiology has the duality of theory and practice. In the cultivation of theoretical knowledge, attention should be paid to the cultivation of practical application capacity. To better construct the curriculum system of spatial epidemiology, further empirical studies are needed in China.

## Conclusion

The study examined the international education trends, the demands of public health fields, and the current situation of spatial epidemiology education in China. We detected huge gaps between the current situation of university education in spatial epidemiology among public health postgraduates and the professional demands of public health fields. To bridge the gaps, and prepare for and respond to a future pandemic of EID, it is urgent to incorporate the teaching and training of spatial epidemiology into the education system of public health postgraduates in China, particularly into the compulsory curriculum system. The next generation of public health talents in China should be well equipped with spatio-temporal thinking to rapidly cope with pandemic.

### Supplementary Information

**Additional file 1: Fig. S1.  **The development situation of public health postgraduate education system in China. **Fig. S2. **The distribution of universities having offered the course of spatial epidemiology, and the proportion of having learned the course of spatial epidemiology among public health postgraduates by provinces in China. **Fig. S3. **The distribution of universities having offered the course of spatial epidemiology, and the median on demand degree of spatial epidemiology among public health postgraduates by provinces in China. 

**Additional file 2: Supplementary Material 1. **Questionnaire for the knowledge, application, and demand for spatial epidemiology among public public health professionals.

**Additional file 3: Supplementary Material 2. **Questionnaire for the learning, application, and demand for spatial epidemiology among public health postgraduates.

**Additional file 4: Supplementary Material 3. **Measurement.

**Additional file 5: Table S1.**Training standards for basic knowledge and capacity of public health postgraduates by international public health education institutions. **Table S2. **The curriculum system for training basic knowledge and capacity of spatial epidemiology of public health postgraduates in top-level universities worldwide. **Table S3. **The median of public health professionals who think it was necessary for public health postgraduates to learn spatial epidemiology at university (median, IQR). **Table S4.** The proportion of having offered the course of spatial epidemiology between national-level universities and non-national-level universities (n, %). **Table S5. **The approaches to learn spatial epidemiology and the statistical software used by public health postgraduates. **Table S6. **The median on the degrees of learning and practical application in spatial epidemiology between public health postgraduates at national-level universities and public health postgraduates at non-national-level universities (median, IQR). **Table S7. **The median on the degrees of learning and practical application in spatial epidemiology between master-degree public health postgraduates and PhD-degree public health postgraduates (median, IQR). **Table S8. **The median on the degrees of learning and practical application in spatial epidemiology between science-degree public health postgraduates and professional-degree public health postgraduates (median, IQR). **Table S9. **The meidan on the degrees of learning and practical application in spatial epidemiology between public health postgraduates majored in different second-level disciplines of public health (median, IQR). **Table S10. **The median on the degrees of learning and practical application in spatial epidemiology between public health postgraduates graded in different academic years (median, IQR). **Table S11. **The learning situation of spatial epidemiology among master-degree public health postgraduates (n, %). **Table S12. **The median on the degree of learning and application in spatial epidemiology between science-degree public health master's postgraduates and professional-degree public health master's postgraduates (median, IQR). **Table S13. **The median on the degrees of learning and application in spatial epidemiology between master-degree postgraduates majored in different second-level disciplines of public health (median, IQR). **Table S14. **The median on the degree of efficacy perception of application of spatial epidemiology to solve public health issues among master-degree public health postgraduates (median, IQR). **Table S15. **The median on the degree of demands in spatial epidemiology among master-degree public health postgraduates (median, IQR). **Table S16. **The demands in offering the course of spatial epidemiology at universities among master-degree public health postgraduates (n, %). **Table S17. **The demands in different contents of spatial epidemiology among public health postgraduates. 

## Data Availability

The anonymized datasets generated or analyzed during this study are available from the corresponding author upon reasonable request. They are not publicly available, as permission was not explicitly sought from survey respondents to use their data in this way.

## Abbreviations

GIS: Geographic Information Science
COVID-19: Corona Virus Disease 2019
SARS-COV: Severe Acute Respiratory Syndrome Coronavirus
MERS-CoV: Middle East Respiratory Syndrome associated Coronavirus

## National-level Universities

PKU: Peking University
THU: Tsinghua University
XMU: Xiamen University
LZU: Lanzhou University
SYSU: Sun yat-sen University
ZZU: Zhengzhou University
HUST: Huazhong University of Science and Technology
WHU: Wuhan University
CSU: Central south University
JLU: Jilin University
SZU: Suzhou University
NCU: Nanchang University
QHU: Qinghai University
SDU: Shandong University
XJU: Xi'an Jiaotong University
FDU: Fudan University
SJTU: Shanghai Jiao Tong University
SCU: Sichuan University
TU: Tibet University
ZU: Zhejiang University

## Non-national-level Universities

AMU: Anhui Medical University
BMU: Bengbu Medical University
CMU: Capital Medical University
FMU: Fujian Medical University
GDUP: Guangdong Pharmaceutical University
GZMU: Guangzhou Medical University
SMU: Southern Medical University
GXMU: Guangxi Medical University
GMU: Guilin Medical University
GMU: Guizhou Medical University
HAINAN MU: Hainan Medical University
HEBEI MU: Hebei Medical University
NCUST: North China University of Technology
XMU: Xinxiang Medical University
HARBIN MU: Harbin Medical University
HBMU: Hubei University of Medicine
WUST: Wuhan University of Science and Technology
NMU: Nanjing Medical University
CHINA MU: China Medical University
IMMU: Inner Mongolia Medical University
NXMU: Ningxia Medical University
SFMU: Shandong First Medical University
WFMU: Weifang Medical University
SXMU: Shanxi Medical University
XNMU: Southwest Medical University
XJMU: Xinjiang Medical University
DLU: Dali University
KMU: Kunming Medical University
NBU: Ningbo University
BTMU: Baotou Medical University
CQMU: Chongqing Medical University
