# Supplementary material for: Self-reported critical gaps in the essential knowledge and capacity of spatial epidemiology between the current university education and competency-oriented professional demands in preparing for a future pandemic among public health postgraduates in China: a nationwide cross-sectional survey
Source: BMC Med Educ. 2023 Sep 7;23:646. doi: 10.1186/s12909-023-04578-6 (PMC10485961; doi:10.1186/s12909-023-04578-6)
Supplement: Supplementary file 1 — Additional file 1: Fig. S1. The development situation of public health postgraduate education system in China. Fig. S2. The distribution of universities having offered the course of spatial epidemiology, and the proportion of having learned the course of spatial epidemiology among public health postgraduates by provinces in China. Fig. S3. The distribution of universities having offered the course of spatial epidemiology, and the median on demand degree of spatial epidemiology among public health postgraduates by provinces in China. [file 12909_2023_4578_MOESM1_ESM.docx]

| **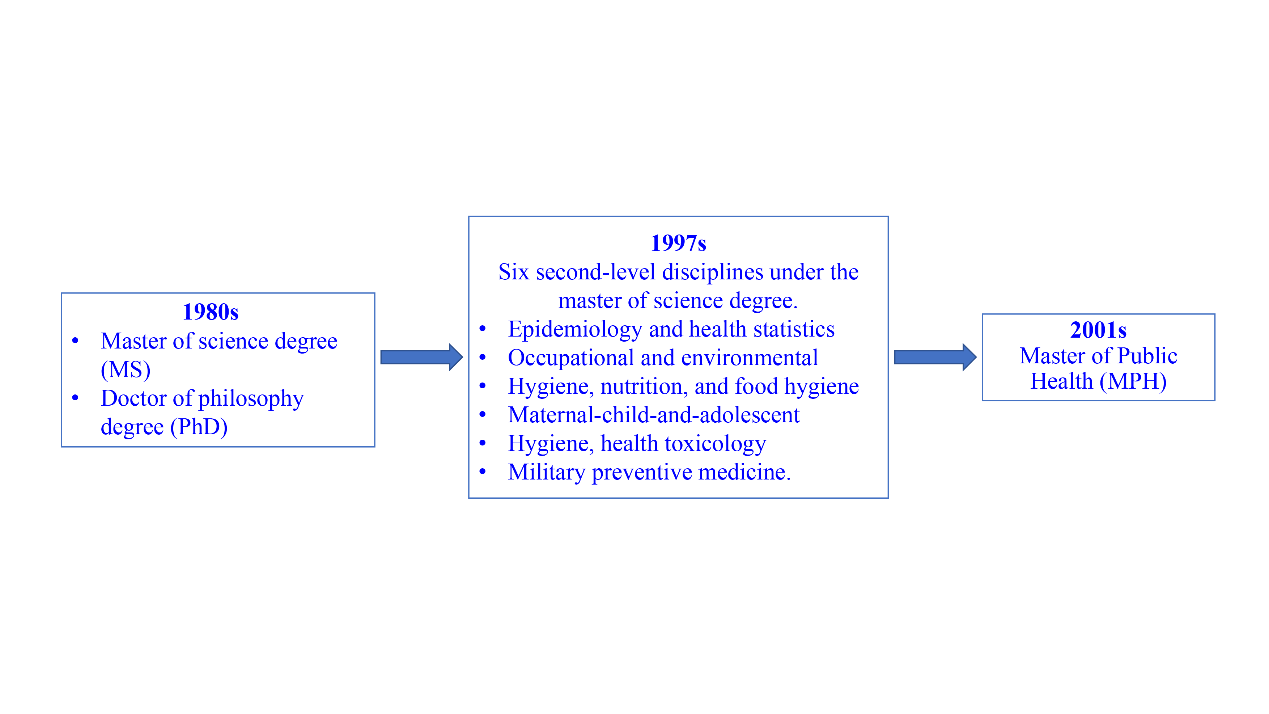** |
| --- |

**Fig. S1** The development situation of public health postgraduate education system in China

**
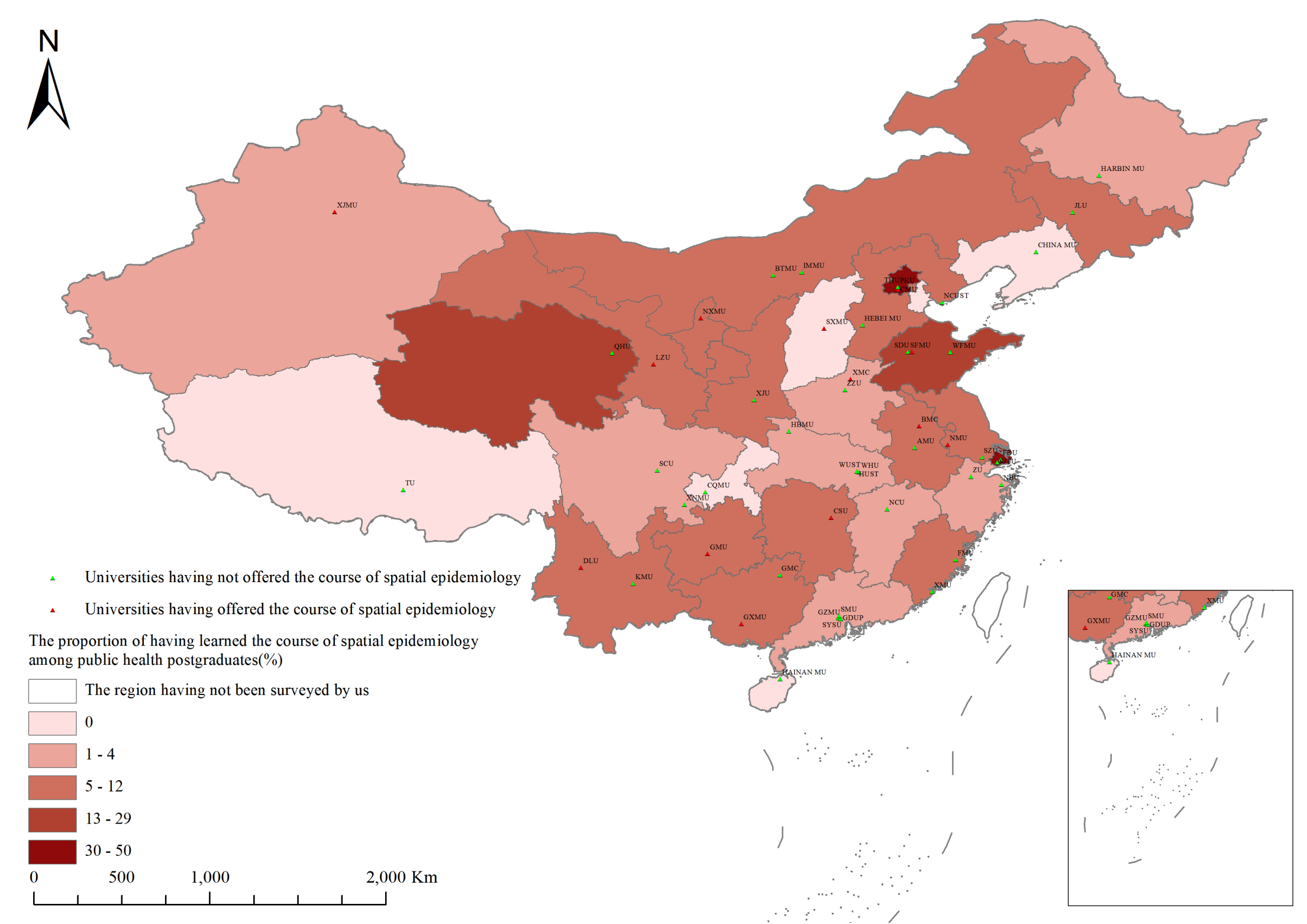
**

**Fig. S2** The distribution of universities having offered the course of spatial epidemiology, and the proportion of having learned the course of spatial epidemiology among public health postgraduates by provinces in China

**
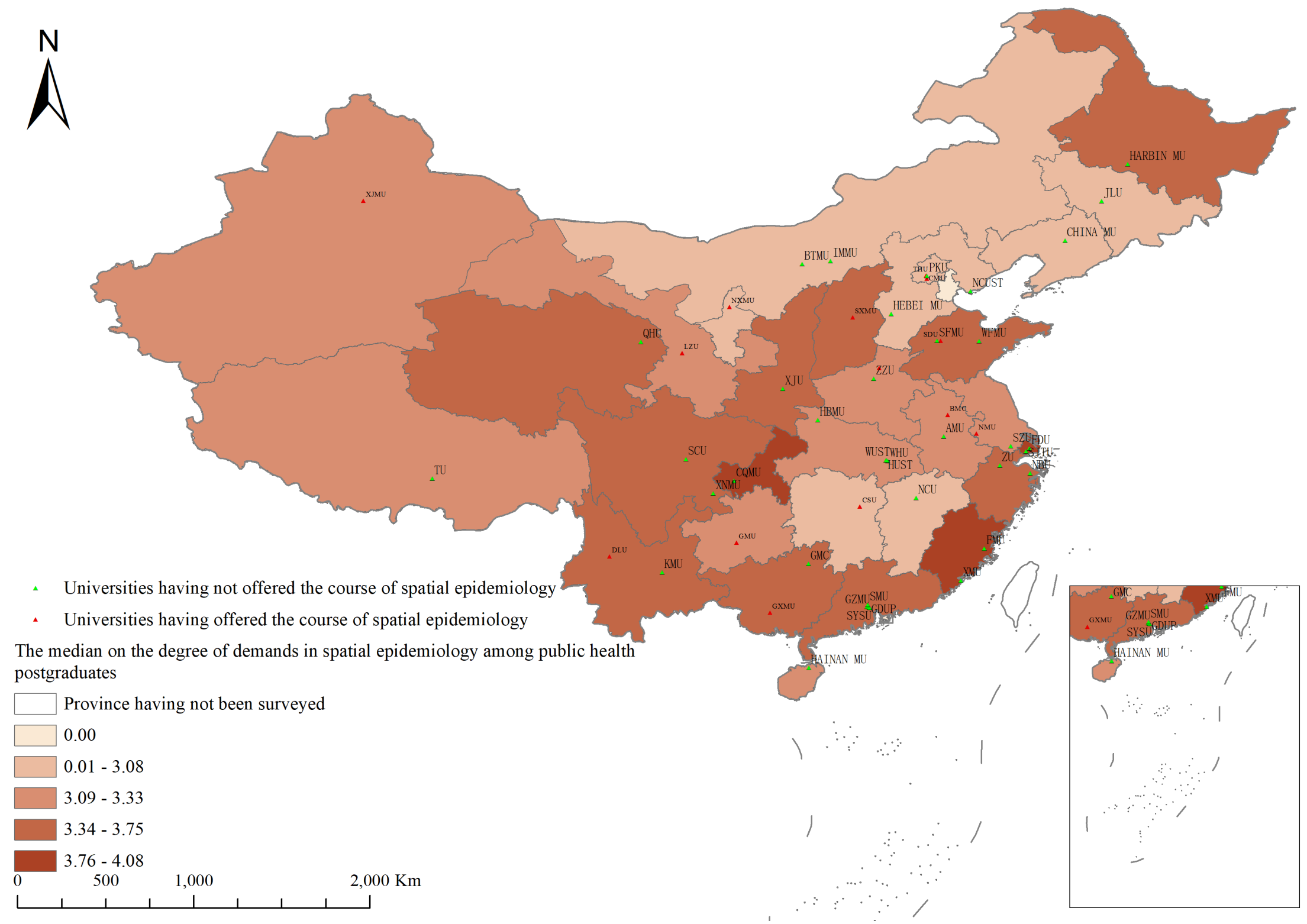
**

**Fig. S3** The distribution of universities having offered the course of spatial epidemiology, and the median on demand degree of spatial epidemiology among public health postgraduates by provinces in China
