# Supplementary material for: Self-reported critical gaps in the essential knowledge and capacity of spatial epidemiology between the current university education and competency-oriented professional demands in preparing for a future pandemic among public health postgraduates in China: a nationwide cross-sectional survey
Source: BMC Med Educ. 2023 Sep 7;23:646. doi: 10.1186/s12909-023-04578-6 (PMC10485961; doi:10.1186/s12909-023-04578-6)
Supplement: Supplementary file 2 — Additional file 2: Supplementary Material 1. Questionnaire for the knowledge, application, and demand for spatial epidemiology among public public health professionals. [file 12909_2023_4578_MOESM2_ESM.docx]

**Supplementary Material 1**

**Questionnaire for the knowledge, application, and demand for spatial epidemiology among public public health professionals**

Dear public health professionals:

Hello! During the COVID-19 pandemic, the relevant theories, methods, and thinking of spatial epidemiology played a role in spatio-temporal trajectory determination, epidemic transmission path tracing, close contactor identification, and other epidemic prevention and control with spatio-temporal big data support. To grasp the current situation of public health professionals’ **knowledge, application, and demand for basic theories, knowledge, methods, and skills of spatial epidemiology**, **the current situation of public health institutions’ talent pool** in the field of spatial epidemiology and spatio-temporal big data, and **the requirements for public health professionals’ competence** in the field of spatial epidemiology and spatio-temporal big data in the new era of public health operational practice. Professor Tang Xiangyan’s group at the School of Public Health, Guangxi Medical University is conducting an online survey. We hope that you will take 6 minutes to fill out the survey. Your participation will help us to optimize the training of public health postgraduates, shape the **spatio-temporal concepts and spatio-temporal thinking** of future public health professionals, and enhance the **spatio-temporal data capabilities** of future public health professionals. Thank you for your support and please receive a small red packet when you submit your questionnaire!

**1. The year you started working in public health:** [Fill in the blank]

**2. Your current workplace is:** [Fill in the blank]

**3. Your job title** [Single choice]

- Junior title
- Intermediate professional title
- Associate senior title
- Senior title

**4. Type of public health practice you are mainly involved in** [Multiple choice]

- Prevention and control of infectious diseases
- Prevention and control of chronic non-communicable diseases
- Public health emergency response
- Immunisation planning
- AIDS prevention and control
- Tuberculosis prevention and control
- Parasitic and endemic disease control
- Health hazards monitoring and control (e.g., occupational health, environmental health, radiological health, school health, etc.)
- Food safety monitoring
- Health education and health promotion
- Other operations:

**5. Your highest level of education** [Single choice]

- Tertiary and below
- Bachelor’s degree
- Master’s degree
- Ph.D. degree

**6. The professional background of your highest qualification** [Single choice]

- Public health and preventive medicine
- Public administration (e.g. health business management)
- Clinical medicine
- Dentistry
- Nursing
- Pharmacy
- Other specialties:

**7. The university from which you graduated with your highest qualification:** [Fill in the blank]

**8. Have you studied spatial epidemiology according to the operational demands of your job?** (Note: Anyone who has attended lectures and training on GIS, spatial analysis techniques, spatio-temporal statistics, spatial epidemiology, etc. can be understood as having "studied") [Single choice]

- Have studied
- Haven't studied yet **(Jump 12)**

**9. In what ways do you learn about spatial epidemiology during your work?** [Multiple choice]

- Academic conferences and academic lectures
- Intensive face-to-face teaching and training
- Further study
- Participate in online courses
- Read literature and publications
- Follow the WeChat public website
- Other pathways:

**10. How well have you learned and mastered the following design methods at the study design level of Spatial Epidemiology?** [Matrix single choice]

| **Item** | **Preliminary learning** | **Systematic learning** | **In-depth learning** |
| --- | --- | --- | --- |
| Spatial sampling theory (e.g., spatial simple random sampling, spatial whole group sampling, spatial systematic sampling, spatial stratified sampling, etc.) |  |  |  |
| Spatial sample size estimation (considering the strength of spatial autocorrelation of diseases on the basis of classical sample estimation) |  |  |  |
| Design principles and design elements of spatial epidemiological research methods (e.g., spatial ecological studies, spatial case-control studies, spatial cohort studies, etc.) |  |  |  |
| Spatial causal inference (e.g., identification and control of spatial epidemiological bias) |  |  |  |

**11. How well have you learned and mastered the following statistical analysis methods for the statistical analysis level of spatio-temporal data in spatial epidemiology?** [Matrix single choice]

| **Item** | **Preliminary learning** | **Systematic learning** | **In-depth learning** |
| --- | --- | --- | --- |
| Methods for mapping and visualising the spatial distribution of diseases (e.g., thematic maps of distribution, spatially interpolated maps, Bayesian smoothed disease mapping, etc.) |  |  |  |
| Disease aggregation detection, methods for identifying spatial and temporal patterns of disease (e.g., spatial autocorrelation analysis, scanning statistical analysis, hot spot and cold spot analysis, etc.) |  |  |  |
| Analysis of factors influencing spatio-temporal patterns of disease (e.g., spatial regression models, geographically weighted regression models, Bayesian spatio-temporal regression models, etc.) |  |  |  |
| Spatio-temporal disease warning and prediction models (e.g., scanning statistical warning models, spatial regression prediction models, Bayesian spatio-temporal prediction models, etc.) |  |  |  |
| Spatial-temporal risk assessment methods for diseases (e.g., geo-detected risk analysis, ecological niche risk assessment models, etc.) |  |  |  |
| Spatio-temporal trajectory analysis, epidemic transmission path tracing, risk traceability analysis (e.g., spatio-temporal trajectory analysis, linear directional mean analysis, etc.) |  |  |  |
| Health service (spatial) accessibility and regional equity assessment methods (e.g. two-step moving search method (2SFCA), etc) |  |  |  |
| Spatial analysis methods for remote sensing (RS) data |  |  |  |

**12. According to the operational demands of public health, have you ever applied the relevant theories, methods and thinking of spatial epidemiology to solve practical problems in public health?** (Note: e.g., using spatio-temporal data analysis software such as ArcGIS, GeoDa, Satscan, etc. to detect spatial aggregation of diseases and produce maps of spatial distribution of diseases, etc.) [Single choice]

- Have used in practice
- Not yet in practice **(Jump 17)**

**13 How often do you use spatial epidemiology in your study design work?** [Matrix single choice]

| **Item** | **Never** | **Occasionally** | **Sometimes** | **Often** | **Always** |
| --- | --- | --- | --- | --- | --- |
| Spatial sampling (e.g., spatial simple random sampling, spatial whole group sampling, spatial systematic sampling, spatial stratified sampling, etc.) |  |  |  |  |  |
| Spatial sample size design (Considering the strength of spatial autocorrelation of diseases on the basis of classical sample estimation) |  |  |  |  |  |
| Spatial epidemiological research methods for scientific design (e.g., spatial ecological niche studies, spatial case-control studies, spatial cohort studies, etc.) |  |  |  |  |  |
| Spatial causal inference (e.g., identification and control of spatial epidemiological bias) |  |  |  |  |  |

**14. How often do you use spatial epidemiology in the analysis of spatio-temporal data?** [Matrix single choice]

| **Item** | **Never** | **Occasionally** | **Sometimes** | **Often** | **Always** |
| --- | --- | --- | --- | --- | --- |
| Methods for mapping and visualising the spatial distribution of diseases (e.g., thematic maps of distribution, spatially interpolated maps, Bayesian smoothed disease mapping, etc.) |  |  |  |  |  |
| Disease aggregation detection, methods for identifying spatial and temporal patterns of disease (e.g. spatial autocorrelation analysis, scanning statistical analysis, hot spot cold spot analysis, etc.) |  |  |  |  |  |
| Methods for analysing factors influencing spatio-temporal patterns of disease (e.g., spatial regression models, geographically weighted regression models, Bayesian spatio-temporal regression models, etc.) |  |  |  |  |  |
| Methods for constructing spatio-temporal disease warning and prediction models (e.g., scanning statistical warning models, spatial regression prediction models, Bayesian spatio-temporal prediction models, etc.) |  |  |  |  |  |
| Spatio-temporal risk assessment methods for diseases: (e.g. geo-detected risk analysis, ecological niche risk assessment models, etc.) |  |  |  |  |  |
| Spatio-temporal trajectory analysis, epidemic transmission path tracing, risk traceability analysis methods: (e.g., spatio-temporal trajectory analysis, linear directional mean analysis, etc.) |  |  |  |  |  |
| Health service (spatial) accessibility, health service regional equity assessment methods: (e.g., two-step moving search method (2SFCA), etc.) |  |  |  |  |  |
| Spatial analysis methods for remote sensing (RS) data |  |  |  |  |  |

**15. How often do you use the following spatio-temporal statistical analysis software when conducting statistical analysis of spatio-temporal data?** [Matrix single choice]

| **Item** | **Never** | **Occasionally** | **Sometimes** | **Often** | **Always** |
| --- | --- | --- | --- | --- | --- |
| ArcGIS |  |  |  |  |  |
| QGIS |  |  |  |  |  |
| MapInfo |  |  |  |  |  |
| R |  |  |  |  |  |
| Stata |  |  |  |  |  |
| WinBUGS/OpenBUGS/GeoBUGS |  |  |  |  |  |
| GeoDa/OpenDa |  |  |  |  |  |
| SpaceStat |  |  |  |  |  |
| Satscan/FleXScan |  |  |  |  |  |
| GWR/GWR4/MGWR/GWmodelS |  |  |  |  |  |
| Geographic Detector |  |  |  |  |  |
| SSSI |  |  |  |  |  |
| ENVI |  |  |  |  |  |

**16. What types of practical public health problems do you apply the theory, methods and thinking of spatial epidemiology to?** [Multiple choice]

- Mapping and visualisation of spatial distribution of diseases
- Detection of disease aggregation (hot or cold spots), identification of spatial and temporal patterns of disease
- Analysis of factors influencing spatio-temporal patterns of disease
- Spatio-temporal disease warning forecasting
- Spatio-temporal risk assessment of diseases
- Spatio-temporal trajectory analysis, epidemic transmission path tracing, risk traceability analysis
- Assessment of (spatial) accessibility of health services and regional equity
- Spatial analysis of remote sensing (RS) data
- Other issues:

**17. In the context of your work, to what extent do you think that the theory, methods and thinking of spatial epidemiology play a role in supporting the solution of operational public health problems in the following areas?** [Matrix single choice]

| **Item** | **No**  **effect** | **Small effect** | **Moderate effect** | **Large effect** | **Very large effect** |
| --- | --- | --- | --- | --- | --- |
| Issues in the field of environment and health |  |  |  |  |  |
| Issues in the field of infectious disease epidemiology |  |  |  |  |  |
| Issues in the field of chronic non-communicable disease epidemiology |  |  |  |  |  |
| Issues in the area of essential public health services (e.g., immunisation) |  |  |  |  |  |
| Issues in public health surveillance |  |  |  |  |  |
| Issues in the area of health emergency response |  |  |  |  |  |

**18. To what extent do you demand the theory, methods, skills and thinking of spatial epidemiology in order to solve operational public health problems?** [Single choice]

- No demand
- Little demand
- Moderate demand
- High demand
- Very high demand

**19. What is your level of demand for the theoretical knowledge, methodological skills and thinking concepts of the following modules of spatial epidemiology in order to reserve and improve the spatio-temporal data competencies needed for the new era of public health operations?** [Matrix single choice]

| **Item** | **No demand** | **Little demand** | **Moderate demand** | **High demand** | **Very high demand** |
| --- | --- | --- | --- | --- | --- |
| Disease spatial distribution mapping and visualisation techniques (e.g., disease distribution thematic maps, disease spatial interpolation maps, Bayesian smoothing disease mapping, etc.) |  |  |  |  |  |
| Disease aggregation detection and identification of spatial and temporal patterns of disease (e.g., spatial autocorrelation analysis, statistical analysis of spatial and temporal scans, hot and cold spot analysis, etc.) |  |  |  |  |  |
| Analysis of factors influencing spatio-temporal patterns of disease: (e.g., spatial regression models, geographically weighted regression models, Bayesian spatio-temporal regression models, etc.) |  |  |  |  |  |
| Spatio-temporal warning prediction of diseases (e.g., spatio-temporal scanning statistical warning models, spatio-temporal regression prediction models, Bayesian spatio-temporal prediction models, etc.) |  |  |  |  |  |
| Spatio-temporal risk assessment of diseases (e.g., geo-detected risk analysis, ecological niche risk assessment models, hierarchical Bayesian spatio-temporal models, etc.) |  |  |  |  |  |
| Spatio-temporal trajectory analysis, epidemic transmission path tracing, epidemic risk tracing: (e.g., spatio-temporal trajectory analysis, linear directional mean analysis, etc.) |  |  |  |  |  |
| Health service (spatial) accessibility and regional equity assessment (e.g., two-step moving search method (2SFCA), etc.) |  |  |  |  |  |
| Spatial analysis methods for remote sensing (RS) data |  |  |  |  |  |

**20. How do you think spatio-temporal (trajectory) big data can support outbreak prevention and control (e.g. the new crown pneumonia outbreak)?** [Single choice]

- No effect
- Small effect
- Moderate effect
- Large effect
- Very large effect

**21 To what extent do you think that the theory, methods, skills and thinking of spatial epidemiology are demanded to support the conduct of disease prevention and control operations?** [Single choice]

- No demand
- Little demand
- Moderate demand
- High demand
- Very high demand

**22 Do you think it is necessary for public health postgraduates to study the theory, methods and skills related to spatial epidemiology during their studies in order to respond to the next emerging infectious disease pandemic and to improve the talent pool, capacity reserve and emergency response preparedness?** [Single choice]

- Necessary
- Uncertain
- Unnecessary

**23. In terms of the methodological aspects of study design in spatial epidemiology, how necessary do you think it is for postgraduate public health students to study the following modules during their studies?** [Matrix single choice]

| **Item** | **Very unnecessary** | **Not really necessary** | **Moderate necessary** | **Large necessary** | **Very necessary** |
| --- | --- | --- | --- | --- | --- |
| Spatial sampling (e.g., spatial simple random sampling, spatial whole group sampling, spatial systematic sampling, spatial stratified sampling, etc.) |  |  |  |  |  |
| Spatial sample size design (Considering the strength of spatial autocorrelation of diseases on the basis of classical sample estimation) |  |  |  |  |  |
| Spatial epidemiological research methods for scientific design (e.g., spatial ecological niche studies, spatial case-control studies, spatial cohort studies, etc.) |  |  |  |  |  |
| Spatial causal inference (e.g., identification and control of spatial epidemiological bias) |  |  |  |  |  |

**24. In terms of the methodological aspects of spatio-temporal data analysis in spatial epidemiology, how necessary do you think it is for public health postgraduate students to study the following modules during their studies?** [Matrix single choice]

| **Item** | **Very unnecessary** | **Not really necessary** | **Moderate necessary** | **Large necessary** | **Very necessary** |
| --- | --- | --- | --- | --- | --- |
| Methods for mapping and visualising the spatial distribution of diseases (e.g., thematic maps of distribution, spatially interpolated maps, Bayesian smoothed disease mapping, etc.) |  |  |  |  |  |
| Disease aggregation detection, methods for identifying spatial and temporal patterns of disease (e.g. spatial autocorrelation analysis, scanning statistical analysis, hot spot cold spot analysis, etc.) |  |  |  |  |  |
| Methods for analysing factors influencing spatio-temporal patterns of disease (e.g., spatial regression models, geographically weighted regression models, Bayesian spatio-temporal regression models, etc.) |  |  |  |  |  |
| Methods for constructing spatio-temporal disease warning and prediction models (e.g., scanning statistical warning models, spatial regression prediction models, Bayesian spatio-temporal prediction models, etc.) |  |  |  |  |  |
| Spatio-temporal risk assessment methods for diseases: (e.g. geo-detected risk analysis, ecological niche risk assessment models, etc.) |  |  |  |  |  |
| Spatio-temporal trajectory analysis, epidemic transmission path tracing, risk traceability analysis methods: (e.g., spatio-temporal trajectory analysis, linear directional mean analysis, etc.) |  |  |  |  |  |
| Health service (spatial) accessibility, health service regional equity assessment methods: (e.g., two-step moving search method (2SFCA), etc.) |  |  |  |  |  |
| Spatial analysis methods for remote sensing (RS) data |  |  |  |  |  |
