# Supplementary material for: Self-reported critical gaps in the essential knowledge and capacity of spatial epidemiology between the current university education and competency-oriented professional demands in preparing for a future pandemic among public health postgraduates in China: a nationwide cross-sectional survey
Source: BMC Med Educ. 2023 Sep 7;23:646. doi: 10.1186/s12909-023-04578-6 (PMC10485961; doi:10.1186/s12909-023-04578-6)
Supplement: Supplementary file 3 — Additional file 3: Supplementary Material 2. Questionnaire for the learning, application, and demand for spatial epidemiology among public health postgraduates. [file 12909_2023_4578_MOESM3_ESM.docx]

**Supplementary Material 2**

**Questionnaire for the learning, application, and demand for spatial epidemiology among public health postgraduates**

Dear postgraduates:

Hello! In order to better advance the education in the new theory of epidemiology, thank you for taking 5 minutes of your time to fill out the following 18 questionnaire questions. The purpose of the survey is to understand your current learning, application, and demand of the emerging discipline of spatial epidemiology at the level of **study design, spatio-temporal data analysis, and practical application**. Whether you are familiar with spatial epidemiology or not, we welcome your participation in the survey. Your participation will help us to improve our teaching, research, and human resource development in spatial epidemiology and we look forward to your support!

**1. The university you are currently attending:**  [Fill in the blank]

**2. Your current professional discipline of study** [Single choice]

- Public health (i.e., MPH and DrPH)
- Epidemiology and health statistics
- Health toxicology
- Occupational and environmental hygiene
- Nutrition and food hygiene
- Maternal-child-and-adolescent hygiene
- Social medicine and health management
- Other second-level disciplines

**3. Your current degree of postgraduate study** [Single choice]

- Master-degree public health postgraduate
- PhD-degree public health postgraduate

**4. Your current academic year** [Single choice]

- Postgraduate in the first academic year
- Postgraduate in the second academic year
- Postgraduate in the third academic year
- Postgraduate in the fourth or above academic year

**5. Have you studied the relevant contents of spatial epidemiology?** [Single choice]

- Have studied
- Haven't studied yet **(Jump 14)**

**6. How did you learn spatial epidemiology?**  [Multiple choice]

- Attend face-to-face course study
- Take online courses
- Participate in the research group meeting
- Attend academic conferences and lectures
- Attend special training
- Read literatures and books
- Follow the WeChat official account
- Other ways

**7. In terms of theoretical principles at the "study design level", how well you have learned and mastered the following contents of spatial epidemiology?**  [Matrix single choice]

| **Item** | **Preliminary learning** | **Systematic learning** | **In-depth learning** |
| --- | --- | --- | --- |
| Spatial sampling theory |  |  |  |
| Spatial sample size estimation |  |  |  |
| Design principles and design elements of spatial epidemiological research methods (e.g., spatial ecological studies, spatial case-control studies, spatial cohort studies, etc.) |  |  |  |
| Spatial causal inference |  |  |  |

**8. In terms of the methodological techniques of "data analysis level", how well you have learned and mastered the following contents of spatial epidemiology?**  [Matrix single choice]

| **Item** | **Preliminary learning** | **Systematic learning** | **In-depth learning** |
| --- | --- | --- | --- |
| Disease mapping, visualization techniques for disease-related issues |  |  |  |
| Spatio-temporal pattern identification |  |  |  |
| Aggregation detection/spatial cluster analysis |  |  |  |
| Analysis of causal mechanisms of spatio-temporal patterns |  |  |  |
| Spatio-temporal prediction and early warning models |  |  |  |
| Spatio-temporal risk assessment |  |  |  |
| Assessment of accessibility of medical and health services |  |  |  |
| Risk assessment of point or line sources |  |  |  |
| Geographical correlation research |  |  |  |

**9. In terms of the field of "practical application level", how well you have learned and mastered the following contents of spatial epidemiology?** [Matrix single choice]

| **Item** | **Preliminary learning** | **Systematic learning** | **In-depth learning** |
| --- | --- | --- | --- |
| Environmental and health research |  |  |  |
| Research on injury epidemiology |  |  |  |
| Spatio-temporal patterns and determinants of infectious diseases |  |  |  |
| Spatio-temporal patterns and determinants of chronic non-communicable diseases |  |  |  |
| Medical and health services |  |  |  |
| Public health surveillance |  |  |  |

**10. How do you view the role of spatial epidemiology in the "practical application level"?** [Matrix single choice ]

| **Item** | **No**  **effect** | **Small effect** | **Moderate effect** | **Large effect** | **Very large effect** |
| --- | --- | --- | --- | --- | --- |
| Environment and health |  |  |  |  |  |
| Injury epidemiology |  |  |  |  |  |
| Spatio-temporal patterns and determinants of infectious diseases |  |  |  |  |  |
| Spatio-temporal patterns and determinants of chronic noncommunicable diseases |  |  |  |  |  |
| Medical and health services |  |  |  |  |  |
| Public health surveillance |  |  |  |  |  |

**11. How often do you use spatial epidemiology in your study design work?** [Matrix single choice]

| **Item** | **Never** | **Occasionally** | **Sometimes** | **Often** | **Always** |
| --- | --- | --- | --- | --- | --- |
| Spatial sampling |  |  |  |  |  |
| Spatial sample size design |  |  |  |  |  |
| Spatial epidemiological research methods for scientific design |  |  |  |  |  |
| Spatial causal inference (e.g., spatial epidemiological bias) |  |  |  |  |  |

**12. How often do you use spatial epidemiology in the analysis of spatio-temporal data?** [Matrix single choice]

| **Item** | **Never** | **Occasionally** | **Sometimes** | **Often** | **Always** |
| --- | --- | --- | --- | --- | --- |
| Disease mapping, visualization techniques for disease-related issues |  |  |  |  |  |
| Spatio-temporal pattern determination |  |  |  |  |  |
| Aggregation detection |  |  |  |  |  |
| Spatio-temporal pattern genesis identification |  |  |  |  |  |
| Spatio-temporal early warning prediction model construction |  |  |  |  |  |
| Spatio-temporal risk assessment |  |  |  |  |  |
| Assessment of spatial accessibility and equity of medical and health services |  |  |  |  |  |

**13. What statistical software have you used to analyze spatio-temporal data?** [Multiple choice]

- ArcGIS
- QGIS
- GeoDa
- WinBUGS/OpenBUGS
- CrimeStat
- Satscan/FleXScan
- R software
- SAM
- Geographic Detector
- GWR4
- Fragstats
- Maxent/GARP/DIVA-GIS
- Other statistical softwares

**14. What is your degree of knowledge demand for the following study components of spatial epidemiology?** [Matrix single choice]

| **Item** | **No demand** | **Little demand** | **Moderate demand** | **High demand** | **Very high demand** |
| --- | --- | --- | --- | --- | --- |
| Disease mapping, visualization techniques for disease-related problems (e.g., spatial mapping, spatial interpolation, etc.) |  |  |  |  |  |
| Aggregate detection of disease or disease-related factors (e.g., Moran I global autocorrelation, LISA I local autocorrelation, Kulldorff time-scan statistics, etc.) |  |  |  |  |  |
| Pathway tracking and risk tracing of disease or disease-related factors (e.g., geographic probes, trajectory analysis, etc.) |  |  |  |  |  |
| Identification of factors influencing disease or disease-related factors (e.g., spatial regression) |  |  |  |  |  |
| Risk assessment of disease or disease-related factors (e.g., ecological niche model, spatial filter model, Bayesian spatio-temporal multilevel model, etc.) |  |  |  |  |  |
| Spatio-temporal early warning analysis of disease or disease-related factors (e.g., Kulldorff spatio-temporal scanning statistics, Bayesian spatio-temporal models, etc.) |  |  |  |  |  |

**15. Does your university offer a course about spatial epidemiology?** [Single choice]

- Not offered
- Not sure
- Have offered

**16. Do you think it is necessary to set up a course about spatial epidemiology at university?** [Single choice]

- Necessary
- Not sure
- Not necessary

**17. In terms of theoretical principles at the study design level, what would you expect to be taught if a course on spatial epidemiology was to be offered at university?**  [Multiple choice]

- Spatial sampling theory
- Spatial sample size estimation
- Design principles and design elements of spatial epidemiological research methods (e.g., spatial ecological studies, spatial case-control studies, spatial cohort studies, etc.)
- Spatial causal inference
- Other theoretical principles to be taught

**18. In terms of the methods and techniques of spatio-temporal data analysis, what would you expect to be taught if a course on spatial epidemiology was to be offered at university?** [Multiple choice]

- Disease mapping, visualization techniques for disease-related issues
- Spatio-temporal pattern recognition
- Agglomeration detection or spatial clustering analysis of diseases
- Spatio-temporal pattern causation mechanism analysis
- Spatio-temporal prediction and early warning models
- Spatio-temporal risk assessment
- Assessment of accessibility of medical and health services
- Risk assessment of point or line sources
- Geographic correlation study
- Other data analysis methods and techniques to be taught
