# Supplementary material for: Self-reported critical gaps in the essential knowledge and capacity of spatial epidemiology between the current university education and competency-oriented professional demands in preparing for a future pandemic among public health postgraduates in China: a nationwide cross-sectional survey
Source: BMC Med Educ. 2023 Sep 7;23:646. doi: 10.1186/s12909-023-04578-6 (PMC10485961; doi:10.1186/s12909-023-04578-6)
Supplement: Supplementary file 4 — Additional file 4: Supplementary Material 3. Measurement. [file 12909_2023_4578_MOESM4_ESM.docx]

**Supplementary Material 3**

**Measurement**

The learning degree of spatial epidemiology of public health professionals consisted of 12 questions in two subsections, i.e., four questions in subsection of study design theories, and eight questions in subsection of spatio-temporal data analysis methods. All questions in the learning degree of spatial epidemiology were scored on a 3-point Likert scale ranging from 1 (preliminary learning) to 3 (in-depth learning). The total score of the learning degree of spatial epidemiology was the sum of scores of all questions. The total score of each subsection was the sum of score of all questions in this subsection. Specifically, the possible sum scores range from 12 to 36 for the learning degree of spatial epidemiology, 4 to 12 for the subsection of study design theories, and 8 to 24 for the subsection of spatio-temporal data analysis methods. Dividing the sum score by the number of questions, we calculated the average score for the learning degree of spatial epidemiology and each subsection. Thus, the average score for the learning degree of spatial epidemiology ranges from 1 to 3 points. Given the midpoint result being 2 points, the average score > 2 points was taken into a better learning situation of spatial epidemiology. Likewise, the subsections of study design theories and spatio-temporal data analysis methods were calculated in the same way as above.

The practical application degree of spatial epidemiology of public health professionals contained three subsections, i.e., subsections of study design theories (four questions), subsections of spatio-temporal data analysis methods (eight questions), subsections of spatio-temporal statistical analysis software (four questions). Each question was scored on a 5-point Likert scale, ranging from 1 (never use) to 5 (always use). The sum score and average score were calculated similarly to that in the learning degree of spatial epidemiology. The total scores ranged from 25 to 125 for the practical application degree of spatial epidemiology, 4 to 20 for subsection of study design theories, 8 to 40 for subsection of spatio-temporal data analysis methods, and 13 to 65 for subsection of spatio-temporal statistical analysis software. The average score for the practical application degree of spatial epidemiology ranges from 1 to 5 points, thus average score > 3 points represented a high frequency of application in spatial epidemiology.

For the public health professional efficacy perceptions of public health professionals of application of spatial epidemiology to solve public health issues, there were 6 questions. A 5-point Likert scale was used, where 1 point represented having no effect and 5 points represented having a very large effect. The calculation approaches for the sum score and average score were similar to that in the learning degree of spatial epidemiology. For this subsection, the total scores range from 6 to 30 points, and the average score ranges from 1 to 5 points. Since the midpoint result was 3 points, an average score > 3 points were viewed as having large to a very large effect on spatial epidemiology in solving public health issues.

A total of 8 questions were selected to evaluate the professional demands in spatial epidemiology among public health professionals. We used the 5-point Likert scale to rate their demands from 1 (no demand) to 5 (very great demand). For this subsection, the total scores range from 8 to 40 points. The average score ranges from 1 to 5 points the average score > 3 points represented having great to very great demands in spatial epidemiology among public health professionals.

For public health professional perceptions of public health postgraduates learning about spatial epidemiology, there were two subsections, i.e., subsections of study design theories (four questions), and subsections of spatio-temporal data analysis methods (eight questions). Each question was scored on a 5-point Likert scale, ranging from 1 (very unnecessary) to 5 (very necessary). The total scores ranged from 12 to 60 for the necessary degree for public health postgraduates to study spatial epidemiology, 4 to 20 for subsection of study design theories, and 8 to 40 for subsection of spatio-temporal data analysis methods. The average score ranges from 1 to 5 points, thus average score > 3 points represented a high necessary degree to learn spatial epidemiology.

The learning degree of spatial epidemiology of public health postgraduates consisted of 19 questions in three subsections, i.e., four questions in subsection of study design theories, nine questions in subsection of spatio-temporal data analysis methods, and six questions in subsection of practical application. All questions in the learning degree of spatial epidemiology were scored on a 3-point Likert scale ranging from 1 (preliminary learning) to 3 (in-depth learning). The possible sum scores range from 19 to 57 for the learning degree of spatial epidemiology, 4 to 12 for the subsection of study design theories, 9 to 27 for the subsection of spatio-temporal data analysis methods, and 6 to 18 for the subsection of practical application. The average score ranges from 1 to 3 points, thus average score > 2 points was taken into a better learning situation of spatial epidemiology. Likewise, the subsections of study design theories, spatio-temporal data analysis methods, and practical applications were calculated in the same way as above.

The practical application degree of spatial epidemiology of public health postgraduates contained two subsections, i.e., subsections of study design theories (four questions), and subsections of spatio-temporal data analysis methods (seven questions). Each question was scored on a 5-point Likert scale, ranging from 1 (never use) to 5 (always use). The total scores ranged from 11 to 55 for the practical application degree of spatial epidemiology, 4 to 20 for subsection of study design theories, and 7 to 35 for subsection of spatio-temporal data analysis methods. The average score ranges from 1 to 5 points, thus average score > 3 points represented a high frequency of application in spatial epidemiology.

For the public health postgraduates’ efficacy perceptions of application of spatial epidemiology to solve public health issues, there were 6 questions. A 5-point Likert scale was used, where 1 point represented having no effect and 5 points represented having a very large effect. The calculation approaches for the sum score and average score were similar to that in the learning degree of spatial epidemiology. For this subsection, the total scores range from 6 to 30 points, and the average score ranges from 1 to 5 points. Since the midpoint result was 3 points, the average score > 3 points were viewed as having a large to very large effect on spatial epidemiology on solving public health issues.

A total of 6 questions were selected to evaluate the potential demands in spatial epidemiology among public health postgraduates. We used the 5-point Likert scale to rate their demands from 1 (no demand) to 5 (very great demand). For this subsection, the total scores range from 6 to 30 points, and the average score ranges from 1 to 5 points. The average score > 3 points represented having great to very great demands in spatial epidemiology among public health postgraduates.
