# Supplementary material for: Self-reported critical gaps in the essential knowledge and capacity of spatial epidemiology between the current university education and competency-oriented professional demands in preparing for a future pandemic among public health postgraduates in China: a nationwide cross-sectional survey
Source: BMC Med Educ. 2023 Sep 7;23:646. doi: 10.1186/s12909-023-04578-6 (PMC10485961; doi:10.1186/s12909-023-04578-6)
Supplement: Supplementary file 5 — Additional file 5: Table S1.Training standards for basic knowledge and capacity of public health postgraduates by international public health education institutions. Table S2. The curriculum system for training basic knowledge and capacity of spatial epidemiology of public health postgraduates in top-level universities worldwide. Table S3. The median of public health professionals who think it was necessary for public health postgraduates to learn spatial epidemiology at university (median, IQR). Table S4. The proportion of having offered the course of spatial epidemiology between national-level universities and non-national-level universities (n, %). Table S5. The approaches to learn spatial epidemiology and the statistical software used by public health postgraduates. Table S6. The median on the degrees of learning and practical application in spatial epidemiology between public health postgraduates at national-level universities and public health postgraduates at non-national-level universities (median, IQR). Table S7. The median on the degrees of learning and practical application in spatial epidemiology between master-degree public health postgraduates and PhD-degree public health postgraduates (median, IQR). Table S8. The median on the degrees of learning and practical application in spatial epidemiology between science-degree public health postgraduates and professional-degree public health postgraduates (median, IQR). Table S9. The meidan on the degrees of learning and practical application in spatial epidemiology between public health postgraduates majored in different second-level disciplines of public health (median, IQR). Table S10. The median on the degrees of learning and practical application in spatial epidemiology between public health postgraduates graded in different academic years (median, IQR). Table S11. The learning situation of spatial epidemiology among master-degree public health postgraduates (n, %). Table S12. The median on the degree of [file 12909_2023_4578_MOESM5_ESM.docx]

**Table S1** Training standards for basic knowledge and capacity of public health postgraduates by international public health education institutions

| **International public health education institutions** | **Public health competences programme** | **Contents** | **Competences** | **Standards for training basic knowledge and capacity on spatial epidemiology** |
| --- | --- | --- | --- | --- |
| ASPHER  (Association of Schools of Public Health in the European Region) | European Core Competences for Public Health Professionals | 1. Methods in public health | (1) Health; (2) Public health; (3) Philosophy of science ; (4) Epidemiology, demography and statistics; (5) Sociology, social psychology and anthropology, etc. |  |
|  |  | **2. Population health and its social and economic determinants** | **(1) Based on information from epidemiological surveillance systems.** | **①Using GIS to produce forecasts for the development of the health status of European populations and population groups;**②Identify, retrieve and analyse major trends of social change;③Identify population groups with elevated health risks. |
|  |  | 3. Population health and its material - physical, | (1) Monitor and interpret environmental exposures; (2) Perform risk assessment associated with components of the physical, radiological, chemical and biological environment, including the likely effect of future climate Change; (3) Develop public health strategies, etc. |  |
|  |  | 1. Health policy; economics; organisational theory and management | (1) Develop and implement a public health strategy based on standard public health methods and guidelines; (2) Perform an organisational, managerial and financial analysis concerning; (3) Perform a health economic assessment of a given procedure, intervention, strategy or policy, etc. |  |
|  |  | 1. **Health promotion: health education, health protection and disease prevention** | (1) Identify population health challenges relevant for health promotion at various levels of social and political organisation, from global to local; **(2) Communicate effectively public health messages–including risk analysis - to lay, professional, academic and political audiences, by use of modern media;** (3) Write a policy proposal, etc. | ①written media and audio-visual techniques |
|  |  | 6. Ethics | (1) Identify ethical aspects of concrete public health interventions, strategies and policies; (2) Ensure the implementation of basic ethical principles in public health strategy making, etc. |  |
| ASPPH (Association of Schools & Programs of Public Health) | MPH Global Health Concentration  Competencies | 1. Evidence-based approaches to Public Health | (1) Apply epidemiological methods to the breadth of settings and situations in public health practice; (2) Select quantitative and qualitative data collection methods appropriate for a given public health context; **(3) Analyze quantitative and qualitative data using biostatistics, informatics, and software, as appropriate, etc.** | **①Utilize the many global health data sets available in the public domain;** ②Discuss the global burden of disease database. |
|  |  | 1. Public Health & Health Care Systems | (1) Compare the organization, structure and function of health care, public health and regulatory systems across national and international settings; (2) Discuss the means by which structural bias, social inequities and racism undermine health and create challenges to achieving health equity at organizational, community and societal levels. |  |
|  |  | 1. Planning & Management to Promote Health | **(1) Assess population needs, assets and capacities that affect communities’ health;** (2) Apply awareness of cultural values and practices to the design or implementation of public health policies or programs, etc. | ①Conduct formative research; ②Conduct a situation analysis across a range of cultural, economic, and health contexts; ③Conduct a situational analysis; **④Validate the health status of populations using available data, etc.** |
|  |  | 1. Policy in Public Health | (1) Discuss multiple dimensions of the policy-making process, including the roles of ethics and evidence; (2) Propose strategies to identify stakeholders and build coalitions and partnerships for influencing public health outcomes; (3) Advocate for political, social or economic policies and programs that will improve health in diverse populations. |  |
|  |  | 1. Leadership | (1) Apply principles of leadership, governance and management, which include creating a vision, empowering others, fostering collaboration and guiding decision making; (2) Apply negotiation and mediation skills to address organizational or community challenges. |  |
|  |  | 1. Communication | (1) Select communication strategies for different audiences and sectors; (2) Communicate audience-appropriate public health content, both in writing and through oral presentation; (3) Describe the importance of cultural competence in communicating public health content. |  |
|  |  | 1. Interprofessional Practice | (1) Perform effectively on interprofessional teams. |  |
|  |  | 1. Systems Thinking | (1) Apply systems thinking tools to a public health issue. |  |

**Table S2**  The curriculum system for training basic knowledge and capacity of spatial epidemiology of public health postgraduates in top-level universities worldwide

| **University** | **Degree** | **Educational Objectives** | **Competences** | **Curriculum** |
| --- | --- | --- | --- | --- |
| 1. Johns Hopkins University | Master of Applied Science in Spatial Analysis for Public Health | The goal of this activity is for students to synthesize knowledge and skills obtained through coursework in a final project that demonstrates mastery of program competencies, as applied to real-world public health and health care questions. | 1. To interpret and solve epidemiological research problems in public health; 2. Apply measures of morbidity and mortality to the assessment and comparison of the health status of populations; **3. Integrate geographical factors to understand how diseases affect public health;**  **4. To obtain information from spatial data technologies and transfer it to databases suitable for mapping; 5. Mapping and spatial integration of public health-related databases using geographic information systems; 6. Analyze and interpret maps using tools from the field of spatial statistics to describe and explain the distribution of health outcomes in populations; 7. Design and implement spatial analysis protocols for addressing public health issues.** | (1) The role of qualitative methods and science in describing and assessing population health; **(2) Spatial analysis of public health; (3) Spatial data technology for cartographic purposes;** (4) Epidemiology; (5) Public health statistics; **(6) Apply spatial statistics,** etc. |
| 1. London School of Hygiene & Tropical Medicine | MSc Health Data Science | to train a new generation of world-leading health data scientists, to work in both the public and private sector. Teaching will focus on building strong quantitative, computational and practical data management skills, while providing opportunities to develop key professional skills required to be a successful health data scientist. | 1. apply statistical and machine learning approaches to analyse health-related data; 2. acquire the tools and skills to manage very large diverse datasets across healthcare systems; 3 develop the professional skills–including teamwork, project management, and presentation skills–to work as a successful data scientist in the public or private sector; **4. understand the varied roles of a health data scientist within the wider health and health research environment;** 5. learn about the key sources of health data, and the context in which these data are collected, implications of the context on issues such as data quality, accessibility, bias and the appropriateness of use to address specific questions; 6. study the key issues related to ethics, security and information governance. | **(1) Introduction to Health Data Science; (2) Epidemiology for Health Data Science;** **(3) Statistics for Health Data Science;** (4) Analysis of Hierarchical and Other Dependent Data; **(5) Spatial Epidemiology in Public Health Analysis of Electronic Health Records; (6) Survival Analysis and Bayesian Statistics,** etc. |
| 1. Yale University | Master of Public Health Sciences - Health Informatics | to provide comprehensive training in health informatics, balancing knowledge from the fields of information science, clinical informatics, population health informatics, big data science and health policy more generally. Emphasis is placed on students acquiring skills in health informatics. Develop skills and knowledge in big data and informatics for health. | 1. select informatics methods appropriate to a particular public health context; **2. comparing the structure and function of health information systems in regional national and international settings; 3. assess population informatics needs, assets and capabilities that affect community health;** 4. propose strategies to identify stakeholders and build alliances and partnerships that affect public health informatics; 5. communicate public health content in the form of written and oral presentations appropriate to the audience; **6. apply systems thinking tools to public health informatics issues.** | (1) clinical research informatics; **(2) data science and health policy more generally; (3) Bayesian statistics; (4) Data and information visualization; (5) Data analysis and causal inference;** **(6) Big data scientific computing methods**, etc. |
| 1. Harvard University | Master of Science | to contribute to the scientific understanding of public health through original laboratory and/or field research, to critically evaluate scientific literature, and to apply scientific knowledge in real-world settings. | 1. address critical scientific and health challenges that affect the lives of individuals and entire populations; **2. building expertise in an area of specialization;** 3. gaining the perspective of multiple public health disciplines. | (1) Biostatistics; (2) Computational Biology and Quantitative Genetics; (3) Environmental Health  Epidemiology; (4) Global Health and Population; (5) **Health Data Science,** etc. |
| 1. University of Michigan | MSc Epidemiology and Systems Modelling | To prepare students to become highly skilled epidemiologists able to understand and analyse mathematical and statistical models of public health problems. | 1. have the knowledge, skills and practical experience required to become an epidemiologist; **2. be proficient in mathematical modelling, computational science and statistics; 3. Apply these models to real data and problem studies.** | **(1) Key concepts in spatial analysis; (2) Applied spatial modelling;** (3) Applied mediated analysis; **(4) Spatial statistics for epidemiological data; (5) Applied epidemiological analysis for causal inference**, etc. |
| 1. Boston University | Master of Public Health Administration | All students have a comprehensive understanding of key public health concepts and the foundational knowledge and skills needed to be a public health professional in the 21st century. | 1. Critically evaluate public health data for continuous improvement of interventions, programs or policies; 2. Design and evaluate community needs assessments or community engagement studies and make recommendations for integrating multiple sources of evidence and community collaboration; 3. Analyze management tools and techniques to solve problems faced by healthcare organizations; **4. apply analytical methods to assess the impact of public health programmes.** | (1) Scientific approach to environmental health; **(2) Geographic Information System (GIS) for public health;** (3) Public health survey methods; (4) Epidemiological concepts and methods; **(5) Use data to make decisions**. |

**Table S3** The median of public health professionals who think it was necessary for public health postgraduates to learn spatial epidemiology at university (median, IQR)

| **Items** | **Median** | **IQR** | **Full mark** |
| --- | --- | --- | --- |
| **Spatial epidemiology related contents** | 4.00 | 3.86-4.00 | 5.00 |
| Subsection of study design theories | 4.00 | 4.00-4.00 | 5.00 |
| Subsection of spatio-temporal data analysis methods | 4.00 | 3.88-4.00 | 5.00 |

**Table S4** The proportion of having offered the course of spatial epidemiology between national-level universities and non-national-level universities (n, %)

| **Variable** | **Total** | **Status of having offered the course of spatial epidemiology** | | ***P-*value^#^** |
| --- | --- | --- | --- | --- |
|  |  | **Yes** | **No** |  |
| **Levels of universities** | 51 (100.00) | 15 (29.41) | 36 (70.59) |  |
| National-level universities | 20 (39.22) | 4 (20.00) | 16 (80.00) | 0.236 |
| Non-national-level universities | 31 (60.78) | 11 (35.48) | 20 (64.52) |  |

^#^ Frequency and percentage were used for statistical description and the comparison was conducted by Chi-square test

**Table S5** The approaches to learn spatial epidemiology and the statistical software used by public health postgraduates

| **Items** | **Frequency(n)** | **Percentage (%)** |
| --- | --- | --- |
| **Learning approaches** |  |  |
| Attend face-to-face course study | 34 | 31.19 |
| Take online courses | 37 | 33.94 |
| Participate in the research group meeting | 31 | 28.44 |
| Attend academic conferences and lectures | 36 | 33.03 |
| Attend special training | 11 | 10.09 |
| Read literature and books | 62 | 56.88 |
| Follow the WeChat official account | 22 | 20.18 |
| Other ways | 8 | 7.34 |
| **Statistical software used for spatio-temporal data analysis** |  |  |
| R software | 71 | 65.14 |
| ArcGIS | 67 | 61.47 |
| GeoDa | 24 | 22.02 |
| Satscan/FleXScan | 19 | 17.43 |
| QGIS | 12 | 11.01 |

**Table S6** The median on the degrees of learning and practical application in spatial epidemiology between public health postgraduates at national-level universities and public health postgraduates at non-national-level universities (median, IQR)

| **Degree of learning and practical application** | **Total**  **n=109** | **National-level universities**  **n=47** | **Non-national-level universities**  **n=62** | ***P-*value^#^** |
| --- | --- | --- | --- | --- |
| **Degree of learning in different subsections** | 1.05 (1.00-1.29) | 1.05 (1.00-1.32) | 1.00 (1.00-1.26) | 0.657 |
| Subsection of study design theories | 1.00 (1.00-1.25) | 1.00 (1.00-1.00) | 1.00 (1.00-1.50) | 0.062 |
| Subsection of spatio-temporal data analysis methods | 1.00 (1.00-1.28) | 1.00 (1.00-1.33) | 1.00 (1.00-1.14) | 0.111 |
| Subsection of practical application | 1.00 (1.00-1.25) | 1.00 (1.00-1.33) | 1.00 (1.00-1.21) | 0.741 |
| **Degree of applying in different subsections** | 1.91 (1.05-2.78) | 1.55 (1.00-2.55) | 2.00 (1.14-2.87) | 0.457 |
| Subsection of study design theories | 1.75 (1.00-2.50) | 1.25 (1.00-2.25) | 2.00 (1.00-2.50) | 0.229 |
| Subsection of spatio-temporal data analysis methods | 1.86 (1.00-2.93) | 1.71 (1.00-2.71) | 2.00 (1.00-3.00) | 0.645 |

^#^Median and interquartile range (IQR) were used for statistical description and the comparison was conducted by Mann-Whitney U test

**Table S7** The median on the degrees of learning and practical application in spatial epidemiology between master-degree public health postgraduates and PhD-degree public health postgraduates (median, IQR)

| **Degree of learning and practical application** | **Total**  **n=109** | **Master-degree public health postgraduates**  **n=101** | **PhD-degree public health postgraduate**  **n=8** | ***P-*value^#^** |
| --- | --- | --- | --- | --- |
| **Degree of learning in different subsections** | 1.05 (1.00-1.29) | 1.05 (1.00-1.26) | 1.06 (1.00-1.66) | 0.651 |
| Subsection of study design theories | 1.00 (1.00-1.25) | 1.00 (1.00-1.25) | 1.00 (1.00-1.56) | 0.958 |
| Subsection of spatio-temporal data analysis methods | 1.00 (1.00-1.28) | 1.00 (1.00-1.22) | 1.06 (1.00-1.67) | 0.306 |
| Subsection of practical application | 1.00 (1.00-1.25) | 1.00 (1.00-1.17) | 1.09 (1.00-1.46) | 0.438 |
| **Degree of applying in different subsections** | 1.91 (1.05-2.78) | 1.82 (1.00-2.78) | 2.05 (1.14-2.98) | 0.578 |
| Subsection of study design theories | 1.75 (1.00-2.50) | 1.75 (1.00-2.50) | 2.25 (1.06-3.63) | 0.260 |
| Subsection of spatio-temporal data analysis methods | 1.86 (1.00-2.93) | 1.86 (1.00-3.00) | 1.93 (1.18-2.61) | 0.832 |

^#^Median and interquartile range (IQR) were used for statistical description and the comparison was conducted by Mann-Whitney U test

**Table S8** The median on the degrees of learning and practical application in spatial epidemiology between science-degree public health postgraduates and professional-degree public health postgraduates (median, IQR)

| **Degree of learning and practical application** | **Total**  **n=109** | **Science-degree public health postgraduates**  **n=56** | **Professional-degree public health postgraduates**  **n=53** | ***P-*value^#^** |
| --- | --- | --- | --- | --- |
| **Degree of learning in different subsections** | 1.05 (1.00-1.29) | 1.00 (1.00-1.20) | 1.05 (1.00-1.45) | 0.225 |
| Subsection of study design theories | 1.00 (1.00-1.25) | 1.00 (1.00-1.00) | 1.00 (1.00-1.50) | 0.395 |
| Subsection of spatio-temporal data analysis methods | 1.00 (1.00-1.28) | 1.00 (1.00-1.19) | 1.00 (1.00-1.44) | 0.584 |
| Subsection of practical application | 1.00 (1.00-1.25) | 1.00 (1.00-1.17) | 1.00 (1.00-1.50) | 0.236 |
| **Degree of applying in different subsections** | 1.91 (1.05-2.78) | 1.59 (1.09-2.53) | 2.00 (1.00-2.87) | 0.517 |
| Subsection of study design theories | 1.75 (1.00-2.50) | 1.38 (1.00-2.44) | 2.00 (1.00-2.63) | 0.385 |
| Subsection of spatio-temporal data analysis methods | 1.86 (1.00-2.93) | 1.79 (1.04-2.71) | 2.00 (1.00-3.00) | 0.624 |

^#^Median and interquartile range (IQR) were used for statistical description and the comparison was conducted by Mann-Whitney U test

**Table S9** The meidan on the degrees of learning and practical application in spatial epidemiology between public health postgraduates majored in different second-level disciplines of public health (median, IQR)

| **Degree of learning and practical application** | **Total**  **n=109** | **Postgraduates majored in public health**  **n=53** | **Postgraduates in discipline of epidemiology and health statistics**  **n=41** | **Postgraduates in other second-level disciplines of public health**  **n=15** | ***P-*value^#^** |
| --- | --- | --- | --- | --- | --- |
| **Degree of learning** | 1.05 (1.00-1.29) | 1.05 (1.00-1.45) | 1.00 (1.00-1.24) | 1.00 (1.00-1.11) | 0.293 |
| Subsection of study design theories | 1.00 (1.00-1.25) | 1.00 (1.00-1.50) | 1.00 (1.00-1.25) | 1.00 (1.00-1.00) | 0.171 |
| Subsection of spatio-temporal data analysis methods | 1.00 (1.00-1.28) | 1.00 (1.00-1.44) | 1.00 (1.00-1.33) | 1.00 (1.00-1.11) | 0.736 |
| Subsection of practical application | 1.00 (1.00-1.25) | 1.00 (1.00-1.50) | 1.00 (1.00-1.17) | 1.00 (1.00-1.17) | 0.397 |
| **Degree of applying** | 1.91 (1.05-2.78) | 2.00 (1.00-2.86) | 1.45 (1.00-2.64) | 2.00 (1.27-2.36) | 0.667 |
| Subsection of study design theories | 1.75 (1.00-2.50) | 2.00 (1.00-2.63) | 1.00 (1.00-2.38) | 2.00 (1.25-2.50) | 0.507 |
| Subsection of spatio-temporal data analysis methods | 1.86 (1.00-2.93) | 2.00 (1.00-3.00) | 1.00 (1.00-2.79) | 1.86 (1.29-2.57) | 0.741 |

^#^ Median and interquartile range (IQR) were used for statistical description and the comparison was conducted by Kruskal-Wallis test.

**Table S10** The median on the degrees of learning and practical application in spatial epidemiology between public health postgraduates graded in different academic years (median, IQR)

| **Degree of learning and practical application** | **Total**  **n=109** | **Postgraduates in the first academic year**  **n=35** | **Postgraduates in the second academic year**  **n=42** | **Postgraduates in the third or above academic year**  **n=32** | ***P-*value^#^** |
| --- | --- | --- | --- | --- | --- |
| **Degree of learning** | 1.05 (1.00-1.29) | 1.00 (1.00-1.26) | 1.05 (1.00-1.33) | 1.05 (1.00-1.31) | 0.689 |
| Subsection of study design theories | 1.00 (1.00-1.25) | 1.00 (1.00-1.25) | 1.00 (1.00-1.06) | 1.00 (1.00-1.25) | 0.895 |
| Subsection of spatio-temporal data analysis methods | 1.00 (1.00-1.28) | 1.00 (1.00-1.33) | 1.00 (1.00-1.33) | 1.00 (1.00-1.19) | 0.893 |
| Subsection of practical application | 1.00 (1.00-1.25) | 1.00 (1.00-1.17) | 1.00 (1.00-1.21) | 1.00 (1.00-1.46) | 0.577 |
| **Degree of applying** | 1.91 (1.05-2.78) | 2.09 (1.00-3.00) | 1.55 (1.00-2.48) | 1.73 (1.27-2.95) | 0.677 |
| Subsection of study design theories | 1.75 (1.00-2.50) | 2.00 (1.00-2.75) | 1.63 (1.00-2.25) | 1.38 (1.00-2.50) | 0.678 |
| Subsection of spatio-temporal data analysis methods | 1.86 (1.00-2.93) | 2.00 (1.00-3.00) | 1.50 (1.00-2.71) | 1.79 (1.29-2.82) | 0.339 |

^#^ Median and interquartile range (IQR) were used for statistical description and the comparison was conducted by Kruskal-Wallis test.

**Table S11** The learning situation of spatial epidemiology among master-degree public health postgraduates (n, %)

| **Variable** | **Total** | **Status of having learned the course of spatial epidemiology** | | ***P-*value^#^** |
| --- | --- | --- | --- | --- |
|  |  | **Yes** | **No** |  |
| **Master-degree public health postgraduates** | 1250(100.00) | 101(8.08) | 1149(91.92) |  |
| **Types of degrees** |  |  |  |  |
| Science-degree public health postgraduates | 592(47.36) | 48(8.11) | 544(91.89) | 0.972 |
| Professional-degree public health postgraduates | 658(42.64) | 53(8.05) | 605(91.95) |  |
| **Second-level disciplines of public health** |  |  |  |  |
| Master of Public Health (MPH) | 658(52.64) | 53(8.05) | 605(91.95) | **0.023** |
| Epidemiology and health statistics | 292(23.36) | 36(12.32) | 256(87.67) |  |
| Health toxicology | 41(3.28) | 2(4.88) | 39(95.12) |  |
| Occupational and environmental hygiene | 71(5.68) | 3(4.23) | 68(95.77) |  |
| Nutrition and food hygiene | 54(4.32) | 2(3.70) | 52(96.30) |  |
| Maternal-child-and-adolescent hygiene | 22(1.76) | 0(0.00) | 22(100.00) |  |
| Social medicine and health management | 91(7.28) | 4(4.40) | 87(95.60) |  |
| Others second-level disciplines | 21(1.68) | 1(4.76) | 20(95.23) |  |

^#^ Frequency and percentage were used for statistical description and the comparison was conducted by Chi-square test

**Table S12**  The median on the degree of learning and application in spatial epidemiology between science-degree public health master’s postgraduates and professional-degree public health master’s postgraduates (median, IQR)

| **Degree of learning and practical application** | **Total**  **n=101** | **Science-degree public health master’s postgraduates**  **n=48** | **Professional-degree public health master’s postgraduates**  **n=53** | ***P*-value^#^** |
| --- | --- | --- | --- | --- |
| **Degree of learning in different subsections** | 1.05 (1.00-1.26) | 1.00 (1.00-1.16) | 1.05 (1.00-1.45) | 0.155 |
| Subsection of study design theories | 1.00 (1.00-1.25) | 1.00 (1.00-1.00) | 1.00 (1.00-1.50) | 0.382 |
| Subsection of spatio-temporal data analysis methods | 1.00 (1.00-1.22) | 1.00 (1.00-1.11) | 1.00 (1.00-1.44) | 0.378 |
| Subsection of practical application | 1.00 (1.00-1.17) | 1.00 (1.00-1.17) | 1.00 (1.00-1.50) | 0.163 |
| **Degree of applying in different subsections** | 1.82 (1.00-2.77) | 1.50 (1.02-2.52) | 2.00 (1.00-2.86) | 0.404 |
| Subsection of study design theories | 1.75 (1.00-2.50) | 1.25 (1.00-2.25) | 2.00 (1.00-2.63) | 0.217 |
| Subsection of spatio-temporal data analysis methods | 1.86 (1.00-3.00) | 1.64 (1.00-2.82) | 2.00 (1.00-3.00) | 0.567 |

^#^Median and interquartile range (IQR) were used for statistical description and the comparison was conducted by Mann-Whitney U test.

**Table S13**  The median on the degrees of learning and application in spatial epidemiology between master-degree postgraduates majored in different second-level disciplines of public health (median, IQR)

| **Degree of learning and practical application** | **Total**  **n=101** | **Master of Public Health**  **n=53** | **Master-degree postgraduates in discipline of epidemiology and health statistics**  **n=36** | **Master-degree postgraduates in other second-level disciplines of public health**  **n=12** | ***P-*value^#^** |
| --- | --- | --- | --- | --- | --- |
| **Degree of learning in different subsections** | 1.05 (1.00-1.26) | 1.05 (1.00-1.45) | 1.03 (1.00-1.21) | 1.00 (1.00-1.05) | 0.155 |
| Subsection of study design theories | 1.00 (1.00-1.25) | 1.00 (1.00-1.50) | 1.00 (1.00-1.25) | 1.00 (1.00-1.00) | 0.092 |
| Subsection of spatio-temporal data analysis methods | 1.00 (1.00-1.22) | 1.00 (1.00-1.44) | 1.00 (1.00-1.31) | 1.00 (1.00-1.08) | 0.450 |
| Subsection of practical application | 1.00 (1.00-1.17) | 1.00 (1.00-1.50) | 1.00 (1.00-1.17) | 1.00 (1.00-1.08) | 0.209 |
| **Degree of applying in different subsections** | 1.82 (1.00-2.77) | 2.00 (1.00-2.86) | 1.41 (1.00-2.52) | 1.82 (1.27-2.84) | 0.564 |
| Subsection of study design theories | 1.75 (1.00-2.50) | 2.00 (1.00-2.63) | 1.00 (1.00-2.25) | 1.75 (1.06-2.00) | 0.391 |
| Subsection of spatio-temporal data analysis methods | 1.86 (1.00-3.00) | 2.00 (1.00-3.00) | 1.50 (1.00-2.82) | 1.93 (1.32-2.89) | 0.659 |

^#^ Median and interquartile range (IQR) were used for statistical description and the comparison was conducted by Kruskal-Wallis test.

**Table S14** The median on the degree of efficacy perception of application of spatial epidemiology to solve public health issues among master-degree public health postgraduates (median, IQR)

| **Variable** | **n (%)** | **Median (IQR)** | ***P*-value** |
| --- | --- | --- | --- |
| **Master-degree public health postgraduates having learned spatial epidemiology** | 101 (100.00) | 4.00 (3.67-4.33) |  |
| **Types of degrees** |  |  |  |
| Science-degree public health postgraduates | 48 (47.52) | 3.92 (3.67-4.17) | 0.405^#^ |
| Professional-degree public health postgraduates | 53 (52.48) | 4.00 (3.67-4.50) |  |
| **Second-level disciplines of public health** |  |  |  |
| Master of Public Health (MPH) | 53 (52.48) | 4.00 (3.67-4.50) | 0.578^†^ |
| Epidemiology and health statistics | 36 (35.64) | 3.92 (3.50-4.17) |  |
| Other second-level disciplines | 12 (18.88) | 3.92 (3.83-4.29) |  |

^†^ Median and interquartile range (IQR) were used for statistical description and the comparison was conducted by Kruskal-Wallis test

^#^ Median and interquartile range (IQR) were used for statistical description and comparison was conducted by Mann-Whitney U test

**Table S15** The median on the degree of demands in spatial epidemiology among master-degree public health postgraduates (median, IQR)

| **Variable** | **n (%)** | **Median (IQR)** | ***P*-value** |
| --- | --- | --- | --- |
| **Master-degree public health postgraduates** | 1250 (100.00) | 3.33 (2.67-4.00) |  |
| **Types of degrees** |  |  |  |
| Science-degree public health postgraduates | 592 (47.36) | 3.17 (2.33-4.00) | 0.057^#^ |
| Professional-degree public health postgraduates | 658 (52.64) | 3.33 (2.83-4.00) |  |
| **Second-level disciplines of public health** |  |  |  |
| Master of Public Health (MPH) | 658 (52.64) | 3.34 (2.83-4.00) | **0.001^†^** |
| Epidemiology and health statistics | 292 (23.36) | 3.37 (2.83-4.00) |  |
| Other second-level disciplines | 300 (24.00) | 3.07 (2.00-4.00) |  |

^†^ Median and interquartile range (IQR) were used for statistical description and the comparison was conducted by Kruskal-Wallis test

^#^ Median and interquartile range (IQR) were used for statistical description and comparison was conducted by Mann-Whitney U test

**Table S16** The demands in offering the course of spatial epidemiology at universities among master-degree public health postgraduates (n, %)

| **Variable** | **Total** | **The necessity of offering the course of spatial epidemiology at universities** | | | ***P-*value^#^** |
| --- | --- | --- | --- | --- | --- |
|  |  | **Necessary** | **Not sure** | **Not necessary** |  |
| **Master-degree public health postgraduates** | 1250 (100.00) | 818 (65.44) | 416 (33.28) | 16 (1.28) |  |
| **Types of degrees** |  |  |  |  |  |
| Science-degree public health postgraduates | 592 (47.36) | 392 (66.22) | 193 (32.60) | 7 (1.18) | 0.842 |
| Professional-degree public health postgraduates | 658 (52.64) | 426 (64.74) | 223 (33.89) | 9 (1.37) |  |
| **Second-level disciplines of public health** |  |  |  |  |  |
| Master of Public Health (MPH) | 658 (52.64) | 426 (64.74) | 223 (33.89) | 9 (1.37) | 0.058 |
| Epidemiology and health statistics | 292 (23.36) | 210 (71.92) | 79 (27.05) | 3 (1.03) |  |
| Health toxicology | 41 (3.28) | 23 (56.10) | 18 (43.90) | 0 (0.00) |  |
| Occupational and environmental hygiene | 71 (5.68) | 48 (67.61) | 21 (29.58) | 2 (2.82) |  |
| Nutrition and food hygiene | 54 (4.32) | 30 (55.56) | 24 (44.44) | 0 (0.00) |  |
| Maternal-child-and-adolescent hygiene | 22 (1.76) | 13 (59.90) | 9 (40.91) | 0 (0.00) |  |
| Social medicine and health management | 91 (7.28) | 60 (65.93) | 29 (31.87) | 2 (2.20) |  |
| Other second-level disciplines | 21 (1.68) | 8 (38.10) | 13 (61.90) | 0 (0.00) |  |

^#^ Frequency and percentage were used for statistical description and the comparison was conducted by Chi-square test

**Table S17** The demands in different contents of spatial epidemiology among public health postgraduates

| **Items** | **Frequency(n)** | **Percentage (%)** |
| --- | --- | --- |
| **The necessity of offering the course of spatial epidemiology at universities** |  |  |
| Necessary | 893 | 65.95 |
| Not sure | 443 | 32.72 |
| Not necessary | 18 | 1.33 |
| **The content demands in subsection of study design theories of spatial epidemiology** |  |  |
| Spatial sampling theory | 696 | 77.94 |
| Spatial sample size estimation | 704 | 78.84 |
| Spatial epidemiological research methods | 853 | 95.52 |
| Spatial causal inference | 752 | 84.21 |
| **The content demands in subsection of spatio-temporal data analysis methods of spatial epidemiology** |  |  |
| Disease mapping and visualization | 826 | 92.50 |
| Spatio-temporal pattern recognition | 588 | 65.85 |
| Cluster detection or spatial cluster analysis | 743 | 83.20 |
| Analysis on the formation mechanism of spatio-temporal pattern | 598 | 66.97 |
| Spatio-temporal prediction and early warning models | 735 | 82.31 |
| Spatio-temporal risk assessment | 656 | 73.46 |
| Evaluation of access to medical and health services | 604 | 67.64 |
| Risk assessment of point or line sources | 501 | 56.10 |
| Geographic correlation study | 506 | 56.66 |
